# Immunomodulatory Mechanism and Potential Application of Dental Pulp-Derived Stem Cells in Immune-Mediated Diseases

**DOI:** 10.3390/ijms24098068

**Published:** 2023-04-29

**Authors:** Qi Min, Liqiong Yang, Hua Tian, Lu Tang, Zhangang Xiao, Jing Shen

**Affiliations:** 1 Laboratory of Molecular Pharmacology, Department of Pharmacology, School of Pharmacy, Southwest Medical University, Luzhou 646000, China; 2 Cell Therapy and Cell Drugs of Luzhou Key Laboratory, Luzhou 646000, China; 3 South Sichuan Institute of Translational Medicine, Luzhou 646000, China

**Keywords:** dental pulp stem cells, immunoregulatory mechanisms, immune-mediated diseases, autoimmune diseases, clinical application

## Abstract

Dental pulp stem cells (DPSCs) are mesenchymal stem cells (MSCs) derived from dental pulp tissue, which have high self-renewal ability and multi-lineage differentiation potential. With the discovery of the immunoregulatory ability of stem cells, DPSCs have attracted much attention because they have similar or even better immunomodulatory effects than MSCs from other sources. DPSCs and their exosomes can exert an immunomodulatory ability by acting on target immune cells to regulate cytokines. DPSCs can also migrate to the lesion site to differentiate into target cells to repair the injured tissue, and play an important role in tissue regeneration. The aim of this review is to summarize the molecular mechanism and target cells of the immunomodulatory effects of DPSCs, and the latest advances in preclinical research in the treatment of various immune-mediated diseases, providing new reflections for their clinical application. DPSCs may be a promising source of stem cells for the treatment of immune-mediated diseases.

## 1. Introduction

Mesenchymal stem cells (MSCs) are widely present in the bone marrow, fat, teeth, umbilical cord, etc., and a small amount is present in peripheral blood [1]. Now it has been found that they can also be isolated and propagated in vitro from the thymus, muscle, pancreas, and lung [2]. Many researchers postulate that perivascular cells are a common origin of MSCs [3,4]. MSCs are a type of stem cells with self-renewal and multi-lineage differentiation potential. Moreover, MSCs play an important role in tissue regeneration and cell therapy. Several studies have shown that MSCs promote myocardial tissue repair in the infarcted heart [5], tissue regeneration in fibrotic disease [6], and are effective in cartilage repair in various osteoarthritis models [7]. In addition, they also promote wound healing [8]. The mechanisms all include targeting MSCs to damaged tissues, differentiating into target cells and promoting tissue regeneration. MSCs have low immunogenicity, allografts do not induce typical immune responses, and their powerful neuromodulatory and immunomodulatory abilities are useful in the treatment of a variety of diseases such as neurodegenerative, autoimmune, and fibrotic diseases [9,10,11]. MSCs are now in mature clinical studies with clinical data demonstrating their safety and efficacy (www.ClinicalTrials.gov, accessed on 25 April 2023). In our study, dental pulp-derived stem cells will be investigated in depth.

Human dental pulp stem cells (hDPSCs) were discovered in 2000 from young impacted molars [12]. They are located within the pulp of human teeth and originate from migrating neural crest cells [13]. They are usually obtained from the permanent teeth of adults [14], and also from the tissue of dead teeth or lesions (such as periodontitis, root resorption, pericoronitis, and osteopetrosis) [15,16,17]. Research has shown that dental pulp stem cells (DPSCs) in the inflamed dental pulp and DPSCs from inherited dental diseases were not damaged by the disease environment and had similar stem cell properties and immunosuppressive effects to healthy DPSCs [17,18,19]. DPSCs have MSCs properties and have the potential to differentiate into osteoblasts, adipocytes, and neuronal cells [20]. Flow analysis has shown that they expressed cluster of differentiation (CD) 10, CD13, CD44, CD90, CD166, CD73, CD29, and CD105, but not CD14, CD15, CD19, CD34, CD45, CD106, CD117, or human leukocyte antigen (HLA)-DR [21]. Compared with other MSCs, DPSCs express low levels of costimulatory molecules, are less immunogenic [22], and have a higher proliferative capacity [23]. Nagpal et al. reported that DPSCs are multipotent stem cells and have limited differentiation potential compared to embryonic or induced pluripotent stem cells, making them potentially safer for clinical use [24]. Because of their wide sources, convenient sampling, and few ethical issues, DPSCs have attracted much attention and research from all walks of life.

When the body is stimulated by antigens, it activates a series of immune responses to protect itself. However, an excessive defense of the body will lead to an imbalance in immunomodulation. In addition, it may cause a series of immune-mediated diseases and inflammatory reactions, such as the most common autoimmune diseases (Sjögren’s syndrome, systemic lupus erythematosus, inflammatory bowel disease, type 1 diabetes, etc.) and the resulting inflammatory reactions. Once the body initiates an autoimmune attack, the persistence of antigens and the recruitment of autoreactive T cells against other autoantigens (antigen and epitope spreading), coupled with low requirements for the costimulatory properties of memory T cells, together promote a vicious cycle of self-maintenance of lifelong disease [25]. Current treatment of these diseases relies on the use of non-antigen-specific, wide-acting immunosuppressive or immunomodulatory compounds that have a wide range of effects and side effects. Although they can reduce autoimmune inflammation, they also impair the normal immune response to pathogens and cancer, and even increase the risk of infections and malignancies [25,26,27]. In recent years, the focus of the treatment of immune-mediated diseases has gradually shifted to cell therapy with low immunogenicity, fewer side effects, and a high cure rate. Thus, MSCs have been widely studied because of their natural and strong immunoregulatory ability [28,29,30].

Although MSCs themselves are not part of the immune system according to existing definitions, they are capable of immunomodulating effects through paracrine or direct contact with other cells (e.g., T cells, B cells, natural killer (NK) cells, macrophages, monocytes, dendritic cells (DCs), neutrophils, etc.) [31,32]. Previous studies have shown that DPSCs exert an immunosuppressive function by inducing the secretion of soluble molecules after interaction with target cells, or by releasing exosomes and extracellular vesicles to function during the stages of development, recruitment, activation, and inhibition of the immune system to regulate the activity of immune cells [33,34,35]. In order to expand the cellular resources for the treatment of immune-mediated diseases, provide data support for clinical treatment, and explore the feasibility of DPSC-mediated therapy in clinical application, it is necessary to understand the immunological characteristics and mechanisms of DPSCs.

## 2. DPSC-Mediated Immune Tolerance

After DPSCs are transplanted into allogeneic organisms, cellular immune rejection will be induced and provoked. In this process, donor cells bind to recipient cells, and this process relies on the recognition of immune-related molecules on the donor cell surface. The major histocompatibility complex (MHC) plays a major role in cellular immune response. MHC class I and MHC class II molecules present endogenous and exogenous antigens to recipient cells, respectively [36]. Compared to somatic cells, DPSCs are less immunogenic, mainly because of low expression levels of MHC class I and negative expression of MHC class II [34]. Therefore, recipient T cells do not readily recognize MSCs and MSCs are able to successfully escape rejection by the host immune system [37], thereby mediating immune tolerance.

## 3. Molecular Mechanisms of Immunoregulatory Function of DPSCs

### 3.1. Soluble Factors Secreted by DPSCs

#### 3.1.1. Transforming Growth Factor Beta (TGF-β) Is a Major Soluble Factor Mediating Immune Tolerance by DPSCs

In a study by Ding et al., through examining the key soluble factors mediating the immunosuppressive function of DPSCs, it was found that TGF-β1 was significantly up-regulated after DPSCs were co-cultured with peripheral blood mononuclear cells (PBMCs) and phytohemagglutinin (PHA) [38]. Moreover, the anti-TGF-β1 monoclonal antibody could restore the proliferation of T cells inhibited by DPSCs, indicating that TGF-β1 is essential in the process of DPSC-mediated immune regulation. Its down-regulation may lead to the inhibition of the immunoregulatory function of DPSCs [38]. This finding was also supported by an investigation by Tomic et al. [39]. In later study, it was found that TGF-β secreted by DPSCs was a major contributor to immunosuppression induced by DPSCs in acute allogeneic immune responses [40]. TGF-β completely abrogated the production of IgM and IgG by allogeneic activation of responder B lymphocytes. Numerous studies have shown that TGF-β secreted by DPSCs can increase CD4^+^CD25^+^Foxp3^+^ regulatory T cells (Tregs) [41] and inhibit the proliferation of allogeneic lymphocytes [42].

#### 3.1.2. Other Soluble Factors

Mesenchymal stem cells secrete many soluble molecules with immunomodulatory effects and act on immune cells. We summarized the immunomodulatory soluble factors secreted by DPSCs from a large number of studies, including interleukin (IL)-6, IL-10, IL-13, IL-29, tumor necrosis factor (TNF-α), macrophage colony stimulating factor (M-CSF), HLA-G, intercellular cell adhesion molecule (ICAM)-1, vascular cell adhesion molecule (VCAM-1), insulin growth factor (IGF)-1, granulocyte macrophage colony stimulating factor (GM-CSF), adiponectin, keratinocyte growth factor, hepatocyte growth factor (HGF), stem cell factor (SCF), vascular endothelial growth factor (VEGF), nitric oxide (NO), and prostaglandin E2 (PGE2) [42,43,44,45,46,47]. These soluble factors are involved in the differentiation and recruitment of lymphocytes and macrophages to varying degrees, as well as the regulation of other related cells, and have made a huge contribution to the immunomodulatory capacity of DPSCs (Table 1).

### 3.2. Role of Indoleamine 2,3-Dioxygenase (IDO)

IDO is a heme-containing cytosolic enzyme that acts as a rate-limiting catalyst in the metabolism of an essential amino acid (tryptophan) of the canine uric acid pathway [18]. IDO degrades the essential amino acid tryptophan into kynurenine, which leads to tryptophan depletion, resulting in the suppression of T-cell proliferation or induction of apoptosis in activated T cells and consequently the induction of tolerance [52]. It has already been reported that the immunosuppressive activity of DPSCs is abolished after the inhibition of IDO-1 expression [39]. In another report, IDO was expressed in DPSCs co-cultured with allogeneic PBMCs and inhibited the proliferation of allogeneic PBMCs for immune regulation [42]. In addition, IDO has also been demonstrated to mediate the inhibitory effect of DPSCs on macrophages. Lee et al. showed that the IDO expression level in DPSCs increased with lipopolysaccharide (LPS) or TNF-α stimulation in a time-dependent manner. The results of co-culture experiments showed that DPSCs can inhibit TNF-α secreted by LPS-triggered macrophages via an IDO-dependent mechanism for immune regulation [18].

### 3.3. Role of Fas/FasL

Activation of the Fas/FasL pathway typically occurs following exposure to an inflammatory microenvironment and induces T cell apoptosis [53]. Mechanistically, upon the binding of FasL to the Fas receptor (CD95), the extrinsic apoptotic pathway is activated, with Pro-Caspase 8 and Fas-associated death domain (FADD) being recruited to form the death-inducing signaling complex (DISC), in which Pro-Caspase 8 undergoes activation. Then, Caspase 8 leaves the DISC, activates caspase 3/7 and induces apoptosis. Alternatively, c-FLIP, a protease-deficient caspase homolog, can interact with FADD and act as an apoptosis inhibitor [54]. MSC-mediated immunotherapy has been found to be associated with FasL expression, and FasL-expressing MSCs are able to induce apoptosis to trigger immune tolerance [55]. Similarly, DPSCs can suppress the activation of allogeneic T lymphocytes by Fas/Fas ligand (Fas/FasL) interaction and the up-regulation of Tregs [56]. In addition, DPSCs improve disease symptoms by expressing FasL in a variety of diseases; for example, knockdown of FasL in DPSCs leads to a reduced ability to improve the colitis phenotype, indicating that FasL is required for DPSC-mediated immune regulation [56]. In another study, DPSCs were able to modulate CD4^+^ T lymphocyte responses in monocytes from patients with primary Sjögren’s syndrome (pSS) by increasing Fas ligand expression [57].

### 3.4. Role of Programmed Cell Death(PD)-1/PD-L1

The PD-1 pathway plays an important role in maintaining central and peripheral immune tolerance [58]. PD-L1 binds to the receptor PD-1 on activated T cells and suppresses antitumor immunity by counteracting T cell-activating signals. Currently, a blockade of PD-1 has been identified as a promising immunotherapeutic approach for cancer and chronic infectious diseases [59]. Previously, it has been found that not only DPSCs express PD-L1 and PD-1, but also PD-1 is important to maintain stem cell properties in hDPSCs [60]. DPSCs can modulate the inflammatory microenvironment by activating PD-1/PD-L1 immunomodulation [61]. When exposed to CD3/CD28-costimulated PBMCs, DPSCs were able to up-regulate PD-L1 through both direct and indirect interaction-dependent mechanisms to suppress immune responses [61]. Pignatti et al. showed that PD-L1 expression was increased in DPSCs after stimulation with activated PBMCs and was involved in the regulation of the immune response, resulting in the increased expression of cleaved caspase3 and decreased expression of IL-2 in PBMCs [62]. The PD-1/PD-L1 signaling pathway is widely involved in a series of processes such as the activation, proliferation, and apoptosis of T cells and inhibits T cell-mediated cellular immune responses [63,64,65]. Further study of the PD-1/PD-L1 signaling pathway may contribute to the clinical application of DPSCs in immune-mediated diseases.

### 3.5. Role of Toll-Like Receptor (TLR) 4

TLR4 is expressed in the odontoblastic cell layer and in areas that associate with blood vessels. When TLR4 is activated, it can regulate the proliferation and migration of DPSCs in deep caries, and it is believed that TLR4 may play an important role in the immune response of DPSCs [66]. The immunomodulatory properties of DPSCs are mentioned to be susceptible to TLR receptor activation, according to a study by Tomic et al. [39]. For example, when DPSCs were exposed to LPS, DPSCs showed enhanced TLR4 expression, mediated increased expression of the anti-inflammatory factor IL-8 through the TLR4 pathway [67], and promoted Wnt5a expression through the TLR4/MyD88/PI3-kinase/AKT pathway [68]. A recent study reported that dental pulp stem cells-derived exosomes (DPSC-exo) could ameliorate cerebral ischemia-reperfusion-induced brain injury in mice, and its anti-inflammatory mechanism may be related to the inhibition of the HMGB1/TLR4/MyD88/NF-κB pathway [69].

### 3.6. Role of PGE2

PGE2 is one of the major effectors of MSC-mediated immunosuppression [70]. PGE2 is a catabolite of arachidonic acid, and during inflammation, PGE2 is thought to act as an anti-inflammatory agent, modulating the inflammatory response and helping to restore tissue homeostasis [71]. PGE2 is an important regulatory molecule that normally synergizes with other immunomodulatory factors such as inducible nitric oxide synthase (iNOS) or IDO to inhibit the proliferation of immune cells and the production of inflammatory factors [72]. DPSCs inhibit the proliferation of allogeneic PBMCs by secreting cytokines such as PGE2 and IL-6 [42]. When stimulated by TNF-α or IL-1β, MSCs secreted significantly more PGE2 [70]. Furthermore, PGE2 has an inhibitory effect on the proliferation of T and NK cells, causes an increase in the pool of Treg, reprograms macrophages to produce the anti-inflammatory cytokine IL-10, and prevents the differentiation of monocytes into DCs [70]. Immunosuppressive factors have been reported to cooperate to exert their regulatory role within inflamed synovium, and PGE2 co-exerts regulatory activity by up-regulating IL-6 [73], reducing local inflammation. It is suggested that PGE2 may play an important role in the immune dysregulation and bone remodeling in the treatment of osteoarthritis by DPSCs [42].

## 4. Immunomodulatory Effects of DPSCs on Immune Cells

### 4.1. Immunomodulatory Effect of DPSCs on Lymphocytes

#### 4.1.1. T Cells

**Figure 1:**
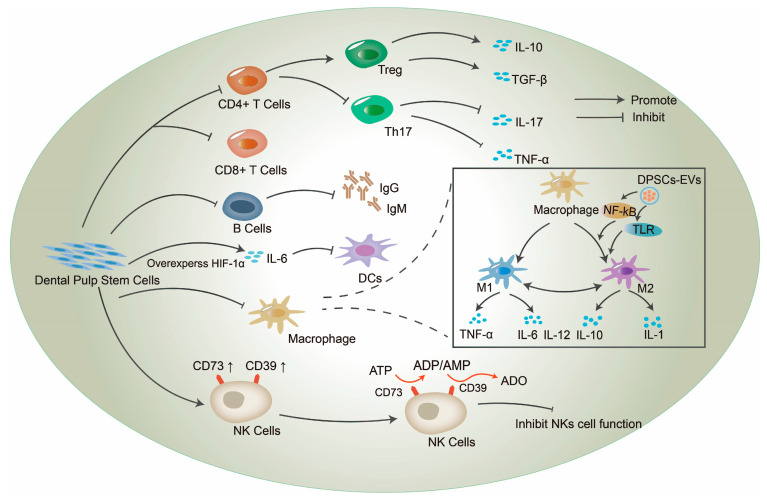
Dental pulp stem cells (DPSCs) act on different target immune cells. DPSCs play an immunomodulatory role by targeting different lymphocytes and antigen-presenting cells. DPSCs regulate the TH17/Treg ratio by inhibiting T lymphocytes, increasing the secretion of anti-inflammatory factors, and decreasing the secretion of pro-inflammatory factors. DPSCs inhibit the function of B lymphocytes and reduce IgG and IgM production, thereby mediating immune tolerance. Moreover, DPSCs also have an inhibitory effect on dendritic cells (DCs) cells and natural killer (NK) cells to regulate the body’s immune function. Among them, DPSCs and their secreted exosomes can transform macrophages from the pro-inflammatory M1 type to the anti-inflammatory M2 type, regulate the secretion of inflammatory factors, and exert their immunomodulatory function.

An increasing number of studies have demonstrated that DPSCs and their derived exosomes have the effect of inhibiting T lymphocyte proliferation in a concentration-dependent manner. For example, in Ding‘s study, PBMCs were stimulated by a T cell mitogen (PHA) or mixed lymphocyte reaction in the presence of DPSCs at the indicated cell ratios (0:1, 0.05:1, 0.2:1,1:1, etc.), and T lymphocyte proliferation was determined [38]. The results showed that all tested cell types significantly inhibited T cell proliferation in different proportions, and in a non-linear manner. Upon challenge with antigens that enter the body, T lymphocytes activate proliferation and differentiate into different subtypes, while releasing different inflammatory factors to exert their effector functions [74]. DPSCs can regulate their activation, proliferation, differentiation, and cytokine secretion, thus affecting the immune response of T cells [43]. Interestingly, the immunosuppressive effect of DPSCs on T cells was not influenced by their differentiation [42], and DPSCs following osteogenic differentiation consistently maintained their immunomodulatory activity [75]. In in vitro co-culture experiments, DPSCs inhibited the proliferation of PHA-stimulated T cells [76], and FasL expressed by DPSCs was able to specifically induce apoptosis in activated T cells [56]. It has also been shown that DPSCs decrease the expression of T cell-specific cytokines interferon-r (IFN-γ) and IL-4, and induce the expression of IDO, PGE2, soluble HLA-G, and HGF [21]. Tregs and Th17 are the most studied T lymphocytes affected by DPSCs. Tregs and Th17 are both CD4^+^ T lymphocytes. Among them, Treg are a class of T cell subsets that control autoimmune reactivity in vivo and play an important role in reducing inflammatory damage and maintaining body self-tolerance [77,78]. Several experiments have shown that DPSCs modulate the Tregs/Th17 ratio (Figure 1). For example, DPSC-exo stimulates CD4^+^ T cells and can inhibit CD4^+^ T cell differentiation into Th17 cells and decrease pro-inflammatory cytokines IL-17 and TNF-α, while promoting CD4^+^ T cell polarization to Treg and increasing the release of cytokines IL-10 and TGF-β [79]. DPSC-driven exosome miR-21 can regulate Treg/Th17 by targeting STAT3 and thus participate in immune responses [80].

#### 4.1.2. B Cells

B lymphocytes produce antibodies and interact closely with T lymphocytes. MSCs interfere with human normal mature B cell function by acting at multiple levels, that is, proliferation, differentiation to antibody-producing cells, and chemotaxis [81]. In addition, the effect of MSCs on B cells is strongly influenced by the relative concentration in vitro. When MSCs and B cells were co-cultured in a ratio of 1:1, the proliferation and differentiation of B cells were most inhibited, and the production of IgM, IgG, and IgA by mature plasma cells was inhibited [82]. However, the inhibitory effect was reduced at lower MSC/B cell ratios [81]. Several studies have demonstrated that bone marrow mesenchymal stem cells (BMSCs) inhibit the proliferation and differentiation of B cells [81,83], and are able to inhibit the terminal differentiation of these cells into plasma cells [83,84]. The immunosuppressive effect of DPSCs on B cells has also been investigated. Furthermore, the study showed that DPSCs suppress allogeneic B cell proliferation, and their capability is higher than BMSCs [38]. At the humoral immune level, DPSCs inhibit the proliferation, antibody production, and differentiation potential of allogeneic T and B cells by releasing TGF-β [49]. Kwack et al.‘s study investigated the mechanism of when mitogen-stimulated B lymphocytes were activated and then released IFN-γ; it was revealed that IFN-γ acted on DPSCs to make them release the soluble factor TGF-β. TGF-β reverses mito-induced IgM and IgG production and inhibits B-lymphocyte function [40].

#### 4.1.3. NK Cells

NK cells are major effector cells of innate immunity and are characterized by the surface expression of the CD56 antigen and the negative expression of CD3. Its function is mainly regulated by signaling balance, through the activation and inhibition of receptors that interact with specific HLA molecules on target cells [85]. NK cells are believed to be important participants in autoimmune diseases and graft-versus-host disease (GVHD). DPSCs can inhibit proliferation and promote apoptosis of activated NK cells [86]. The mechanism is the hydrolysis of adenosine triphosphate (ATP) into adenosine (ADO) by CD39 and CD73 enzymatic activities, thereby inhibiting the function of NK cells [86]. However, the inhibitory effect of MSCs on activated NK cell proliferation varied, depending on the cytokine used to activate the NK cell and the source of MSCs. A study by Najar et al. demonstrated that the proliferation of NK cells activated by IL-2 and IL-15 was slightly decreased by BMSCs [87], and that the proliferation of IL-2, IL-12, IL-15, and IL-21 activated NK cells was partially decreased by adipose mesenchymal stem cells [88]. However, they also showed that the proliferation of NK cells activated by IL-2, IL-12, IL-15, and IL-21 was significantly inhibited by wharton’s jelly mesenchymal stromal cells [89] and foreskin-mesenchymal stromal cells [90]. Studies have shown that the inhibition of MSCs on activated NK cells stimulated by IL-2 is related to the NK/MSC ratio [91]. Likewise, the effect of MSCs on NK cell cytokine production and cytotoxicity were controversial according to different studies [92]. Nonetheless, MSCs have been shown to decrease activating receptors on NK cells and increase inhibitory receptors [92].

On the other hand, NK cells activated by IL-2 have been shown to exert cytotoxicity toward MSCs. IL-2-activated NK cells lysed autogenous and allogeneic MSCs efficiently [93]. Surprisingly, both IL-2-treated or non-treated NK cells could induce death of DPSCs. Interestingly, when DPSCs were co-cultured with monocytes and exposed to IL-2-treated NK cells, NK-mediated cytotoxicity was reduced, partially by competitive lysis of monocytes [94]. When monocytes were co-cultured with DPSCs and removed prior to the interaction of DPSCs with NK cells, the NK cell-mediated lysis was still inhibited. A similar effect was also observed when DPSCs were co-cultured with T and B cells. This suggests a complex interaction of NK cells with MSCs in the presence of other immune effectors, which is not only due to competitive lysis. Although current studies suggest that under inflammatory environments NK cells may be cytotoxic to MSCs, the observations are from in vitro studies. In future, it would be interesting to determine the interaction between NK cells and MSCs in in vivo settings.

### 4.2. Immunomodulatory Effects of DPSCs on Antigen-Presenting Cells

#### 4.2.1. Macrophages

Macrophages are heterogeneous cell populations scattered throughout many tissues and can be divided into two broad categories: classically activated M1 macrophages and alternately activated M2 macrophages. Of these, M1 macrophages are inflammatory cells and M2 macrophages are anti-inflammatory cells [95]. Different pro-inflammatory media will change the polarization state of macrophages (Table 2 and Figure 1). Pro-inflammatory mediators such as LPS and IFN-γ can induce macrophage polarization to the M1 type by activating iNOS, enhancing the production of pro-inflammatory cytokines (TNF-α, IL-6, IL-12), and producing reactive oxygen species, such as NO [96]. On the other hand, macrophages exposed to cytokines such as IL-4 and IL-13 polarize to the M2 type, whereby high levels of cytokines, such as IL-10 and IL-1, are produced [96]. Thus, macrophages show protective or pathogenic effects in a wide range of autoimmune and inflammatory diseases and have strong immunoregulatory functions. Upon stimulation in tissues, macrophages differentiate from monocytes to replace dead macrophages, either to protect tissues from injury, or to repair damaged tissues [95].

Interestingly, DPSCs can regulate macrophage differentiation by secreting soluble factors. Several studies have shown that factors secreted by DPSCs through a paracrine mechanism have a strong M2-inducing activity and can convert the pro-inflammatory M1 environment into the M2 environment. In LPS-pretreated M1-like macrophages, dental pulp stem cells-derived culture medium (DPSC-CM) significantly enhanced the polarization of M1-like macrophages to M2-like macrophages and converted inflammation to anti-inflammation. It increased the number of M2 macrophages by expressing M2-inducing factors such as TNF-α, IL-1β, IL-10 and M-CSF, which can lead to M2 macrophage differentiation or accumulation [46]. Furthermore, extracellular vesicles of dental pulp stem cells (DPSC-EV) transform macrophages to the M2 phenotype by inhibiting TLR and NF-κB signaling, which promotes tooth healing [97]. In addition, DPSCs can also inhibit macrophage function and/or cytokine secretion. It has been reported that DPSCs isolated from both healthy and inflamed pulp tissue can inhibit TNF-α secreted by LPS-triggered macrophages via an IDO-dependent mechanism and inhibit macrophage function [18]. Lee et al. proposed that DPSCs partially block the expression of NF-κB subunit p65, resulting in a decrease in TNF-α production by macrophages, which may be one of the mechanisms by which DPSCs act [18]. Surprisingly, culture medium (CM) derived from stem cells from human exfoliated deciduous teeth (SHED-CM) can secrete a group of M2-like macrophage inducers different from IL-4 and IL-13: monocyte chemoattractant protein-1 (MCP-1) and sectodomain of sialic acid-binding Ig-like lectin-9 (ED-Siglec-9), which can induce macrophage polarization and promote the ability of functional recovery after spinal cord injury [44]. MCP-1/ ED-Siglec-9 induces the pro-inflammatory M1 response and promotes the anti-inflammatory M2 response by decreasing the mRNA expression of pro-inflammatory mediators (TNF-α, IL-1β, IL-6, and iNOS) and increasing the expression of anti-inflammatory M2 markers (TGF-β, IL-10, CD206, and ARG-1) [98]. This is a previously unrecognized set of inducers, and it is notable that ED-Siglec-9 is uniquely present in SHED-CM [44]. Thus, the mechanism by which M1 induces M2 as well as M2-related therapeutic activity varies according to different stem cell types.

#### 4.2.2. DCs

DCs are the most potent professional antigen-presenting cells and play a key role in the effects of immunity and tolerance. In addition to pro-inflammatory effects, DCs also promote immune homeostasis by inducing and maintaining peripheral T-cell tolerance [99], a regulatory process that is also associated with the classical PD-1 axis [65]. MSCs have been shown to exert effects on the differentiation, maturation, and function of different cell populations of DCs. For example, BMSCs significantly affect the ability of DCs to prime T cells [100] and alter the cytokine secretion profile of DCs [101]. BMSCs can cause the mature DC1 type to reduce TNF-α secretion and the mature DC2 type to increase IL-10 secretion [102]. It has been reported that the inhibitory effect of MSCs on DC maturation and function is predicated on their interaction occurring early in the DC maturation process [103]. This suggests that MSCs infusion may be more effective before or immediately after transplantation, for optimal immunosuppressive effect [103]. So far, only one study has reported the effect of DPSCs on DC cells, demonstrating that HIF-1α overexpressing DPSCs is able to effectively impair DC differentiation [28]. Further studies on the interaction between DPSCs and DCs are urgently needed in order to better understand the immunomodulatory effects of DPSCs on DC function.

## 5. Prospective Applications of DPSCs in Immune-Mediated Diseases

DPSCs have been shown to improve a variety of other immune-mediated diseases based on their immunomodulatory function (Table 3). The mechanism involves paracrine and direct cell-to-cell contact to regulate the activation, aggregation, and differentiation of lymphocytes and antigen-presenting cells.

### 5.1. Sjögren’s Syndrome

Sjögren’s syndrome (SS) is a chronic inflammatory autoimmune disease affecting the exocrine glands. Its main symptom is a decrease in water in the mouth and eyes, which causes keratoconjunctivitis sicca and xerostomia [104]. SS is divided into primary Sjögren’s Syndrome (pSS) and secondary Sjögren’s Syndrome (sSS) for different causes of the disease. pSS occurs in the absence of connective tissue disease. sSS is characterized by the presence of other connective tissue diseases, most commonly rheumatoid arthritis, but also systemic sclerosis, systemic lupus erythematosus (SLE), and polymyositis [105]. Although SS shares some features with other systemic autoimmune diseases such as SLE, in terms of activation of innate and adaptive immune pathways, local inflammatory lesions in target organs, and the presence of serum autoantibodies against intracellular components, immunosuppressants and existing biologic agents have not been shown to provide significant therapeutic efficacy [106].

IFN-γ has been shown to be a key factor in the pathogenesis of SS and promotes epithelial cell apoptosis through an extrinsic apoptotic pathway in SS [45,107]. The mechanism is related to IFN-γ-induced increased Fas expression in epithelial cells [108,109]. Various causes also contribute to the pathogenesis of SS, such as the infiltration of T and B cells that activate memory phenotypes and destruction of epithelial cells [110]. With the interaction of various mechanisms, the treatment of SS becomes very difficult. DPSCs, as emerging “immunomodulators”, can effectively inhibit the activation of T and B cells and regulate the Fas/FasL apoptotic pathway, which has been shown to be a potentially effective treatment for SS. Researchers have already conducted research of the treatment of SS with DPSCs. Soluble factors secreted by DPSCs (e.g., TGF-β1, IL-10, and IL-13) can decrease the expression levels of IFN-γ, IL-6, and IL-17a and increase the expression levels of IL-10 and TGF-β1 in the submandibular gland of rats, effectively alleviating the Th17/Treg imbalance [45]. Under this regulation, it can effectively improve the local inflammatory microenvironment, reduce submandibular gland cell apoptosis, and alleviate SS-induced hyposalivation [45]. Another study reported that the direct injection of pulp stem cells from exfoliated deciduous teeth (SHED) into the tail vein of non-obese diabetic (NOD) mice reduced inflammation in the submandibular gland, prevented the deterioration of its apoptosis and autophagy, and restored its secretory function [111]. In a recent study, DPSCs were found to inhibit the proliferation of CD4^+^ T lymphocytes by increasing the expression of the Fas ligand in T lymphocytes and Foxp3 expression in Treg cells and reducing intracellular IFN-γ and IL-17 secretion in SS patients [57]. In the current study, BMSCs are the main source of MSCs for clinical use [112], but studies have shown that DPSCs have a more significant advantage in autoimmune diseases. Compared with BMSCs, DPSCs contain more secreted factors associated with tissue regeneration properties, including cell proliferation, anti-inflammatory effects, and immunomodulatory effects (e.g., TGF-β1, HGF, IL-10, and IL-13) [113]. When comparing DPSCs and BMSCs, DPSCs had a stronger inhibition ability on activated T cells, and the anti-apoptotic ability of DPSCs in SS was greater than that of BMSCs [113], which could effectively relieve the insufficient salivation of SS. In addition, DPSCs can secrete more immunosuppressive factors (e.g., TGF-β1, HGF, IL-10, and IL-13) than BMSCs [113]. When comparing DPSCs and BMSCs, DPSCs showed greater anti-apoptotic capacities in SS than BMSCs [113]. These studies fully demonstrate that DPSCs can effectively combat the pathogenesis of SS and can be used as a potential source of treatment for SS.

### 5.2. Systemic Lupus Erythematosus

SLE is a disease in which various autoantigens seriously affect immune tolerance due to the dysregulation of both the innate and adaptive immune systems. It is caused by excessive B cell and T cell responses and loss of immune tolerance to self-antigens [114]. Defects in antibody production and elimination, circulation and tissue deposition of immune complexes, activation of complement, and cytokines can lead to clinical manifestations ranging from mild fatigue and joint pain to severe life-threatening organ damage [114]. Although SLE is incurable, it can be effectively controlled by medications, including glucocorticoids, antimalarials, nonsteroidal anti-inflammatory drugs, immunosuppressive agents, and B cell-targeted biologics. It is well recognized that taking large amounts of immunosuppressive drugs can have harmful side effects on the body. Furthermore, taking immunosuppressive agents increases the risk of bone marrow suppression in patients, making BMSCs deficient and susceptible to aging and apoptosis in SLE patients. A study comparing BMSCs from SLE patients and normal controls showed that BMSCs in SLE grew slowly, and gradually lost vitality during passage. However, the expression of IL-6 and IL-7 mRNA was obviously down-regulated in MSCs from SLE patients, suggesting that the abnormality of cytokine secretion may led to hematopoietic damage and immune imbalance [115]. BMSCs are not suitable for autologous transplantation at this time, and DPSCs that are not affected by the state of the body can be preferred.

SLE has been found to be associated with an imbalance in the Treg/Th17 ratio [116]. DPSCs play a significant role in regulating the Treg/Th17 ratio in a large number of studies and have great potential for the treatment of SLE. Many in vivo experiments have confirmed the improvement of SLE disease symptoms by DPSCs. For example, in Tang et al.’s study, DPSCs were transferred intravenously into lupus-prone B6/lpr mice. DPSCs were found to reduce 24-h proteinuria levels, anti-dsDNA antibodies, and glomerular IgG/IgM. They can also improve glomerular injury in B6/lpr mice, significantly reduce plasma cells and IFN-γ-producing CD4^+^T cells in the spleen, and alleviate disease symptoms [117]. In a study by Makino et al., it was shown that a systemic infusion of DPSCs can inhibit the hyperactivated T cells in MRL/LPR mice, reduce the overproduction of autoantibodies, and restore renal dysfunction in MRL/LPR mice [30]. SHED exerts immunotherapeutic effects even after cryopreservation. Ma et al. tested the therapeutic efficacy of SHED cryopreserved for over two years in SLE in MRL/lpr mice. Cryopreserved SHED improved SLE symptoms and recovered the increased IL-17-secreting helper T cells in MRL/lpr mice systemically and locally [118]. At the same time, it has been found that the therapeutic effects of single systemic DPSCs might also be mediated via mechanisms other than cell-cell contact. Sonoda et al. reported that SHED-releasing extracellular vesicles (SHED-EVs) contain abundant small RNAs that can promote cell-cell communication [33]. In this study, the different RNA content in SHED-EVs targets the recipient’s BMSCs to promote hematopoietic niche formation and immune regulation by activating Tert-related telomerase activity [33]. In an in vitro assay, SHED-EVs treatment rescued the in vitro immunomodulatory function of BMSCs isolated from MRL/lpr mice, as shown by suppression of induction of CD4^+^IL-17^+^IFN-γ^−^cells and enhancement of induction of CD4^+^CD25^+^Foxp3^+^ and Annexin-V^+^7AAD^+^ cells. These results suggest that SHED-secreted RNAs are important players in treating SLE by systemic transplantation of SHED [33].

### 5.3. Osteoarthritis

Osteoarthritis is one of the most common degenerative joint diseases and is pathologically characterized by the local loss of articular cartilage in synovial joints, with varying degrees of osteophyte formation, subchondral bone changes, and synovitis [119]. Autologous BMSCs have been shown to improve knee arthritis in clinical randomized controlled trials without serious adverse reactions or complications, which is a safe and feasible method [120].

Macrophages play an important role in the progression of osteoarthritis; however, hyperactivated macrophages promote an inflammatory microenvironment [121]. As mentioned earlier, DPSCs regulate macrophage differentiation by secreting soluble factors. hDPSCs have been reported to significantly inhibit osteoarthritic macrophage activation in vitro. In the presence of hDPSCs, osteoarthritic macrophages shifted to a less inflammatory state in terms of cell morphology, immunophenotype, and inflammatory factor expression [48]. In addition, HGF and TGF-β1 are also involved in the DPSC-mediated phagocytosis of macrophages, thereby improving joint pain caused by excessive activation of macrophages to release inflammatory factors [48]. Cui et al. showed that local injection of DPSCs reduced hyperalgesia and synovial inflammation in rats with temporomandibular arthritis and significantly improved their clinical pain and degenerative changes [122]. The mechanism is that DPSCs inhibit the activation of the STAT1 pathway by down-regulating the phosphorylation level of the Tyr 701 site, thereby inhibiting the expression of MMP3 and MMP13 and improving cartilage degradation [122]. Meanwhile, DPSC-CM promoted the survival and proliferation of articular cartilage through secretion-mediated effects [123], and played an anti-apoptotic role in reducing the apoptosis of chondrocytes induced by IL-1β [124]. It has also been pointed out that DPSCs exhibit a high potential to form chondrocytes and osteoblasts and may be useful in the treatment of osteochondral diseases [39]. This has been demonstrated by studies by Lo Monaco et al., where DPSCs differentiated into chondrogenic lineages and could potentially replace damaged cartilage tissue [123]. Because of the anti-inflammatory effects of DPSCs, they also smooth the articular surface of damaged cartilage compared to primary chondrocytes [125].

### 5.4. COVID-19

In recent years, COVID-19 has swept the world and brought different degrees of impact on all people. Pulmonary edema, acute respiratory distress syndrome, and ventilatory dysfunction caused by COVID-19 are caused by the acute release of inflammatory cytokines IL-2, IL-6, IL-7, and TNF-α [126]. MSCs are believed to assist in COVID-19 treatment mainly due to their immunomodulation of immune cells and regeneration of the damaged lung tissues. The immunoregulatory effect of DPSCs can inhibit the secretion of TNF-α through the IDO-mediated pathway, promote the polarization of macrophages, regulate the balance of immune homeostasis, and inhibit the occurrence of inflammation by increasing the secretion of cytokines such as IL-10, PGE2, and IL-6 in COVID-19 [49]. At the level of humoral immunity, DPSCs inhibit the proliferation, antibody production, and differentiation of allogeneic T and B cells by releasing TGF-β1 [49]. DPSCs are considered good candidates for the treatment of COVID-19 due to their powerful immunosuppressive and immunomodulatory functions [49]. Croci et al. investigated the cytokine release of PBMCs from COVID-19 patients alone or co-cultured with DPSCs. They found that compared with healthy patients, activated PBMCs from COVID-19 patients released fewer IL-10 and more IL-18. Moreover, in activated PBMCs, co-culture with DPSCs increased IL-6 and GM-CSF and decreased IFN-γ, TNF-α, IL-2, IL-5, IL-9, IL-10, IL-12, IL-17A, IL-18, IL-21, IL-23, and IL-27 levels [127]. Ye et al. aimed at evaluating the efficacy and safety of DPSCs in the treatment of COVID-19 in a Phase I/II randomized controlled trial, which is still recruiting patients and will provide new reflections on the use of DPSCs in the clinical treatment of COVID-19 [128].

### 5.5. Inflammatory Bowel Disease

Inflammatory bowel disease (IBD) is a general term for a group of chronic, nonspecific inflammatory diseases of the intestine, whose etiology has not been fully elucidated. It mainly includes Crohn’s disease (CD) and ulcerative colitis (UC) [129]. Current studies have shown that the disease is associated with various factors such as genetics, environment, infection, and immunity [129]. The pathogenesis may be that individuals with genetic susceptibility have dysregulation of the normal intestinal flora under the action of various pathogenic factors, causing abnormal intestinal immune responses, resulting in non-specific inflammatory responses in the intestinal mucosa and submucosa [129]. UC is a chronic IBD characterized by superficial mucosal inflammation, rectal bleeding, diarrhea, and abdominal pain [130]. Li et al. provided direct evidence of the protective role of DPSCs in UC [50]. In their study, the HGF gene of a rat was transfected into DPSCs to constitute DPSCs overexpressing HGF (HGF-DPSCs). By exploring the potential mechanism of DPSCs and HGF-DPSCs on UC, it was found that DPSCs could promote the repair of damaged tissues [50]. Furthermore, HGF-DPSCs could differentiate into intestinal stem cell-like cells and promote the proliferation of intestinal stem cell-like cells. They also significantly reduced the expression levels of inflammatory cytokines TNF-α and INF-γ and increased the expression levels of cytokines TGF-β and IL-10, thereby inhibiting the inflammatory response, reducing oxidative stress, and alleviating colon injury [50]. Zhao et al. demonstrated that FasL expressed by DPSCs was essential to induce T cell apoptosis and ameliorate the lesion phenotype in mice with colitis [56]. Pathologic features of CD include transmural inflammation, lymphocytic aggregates, and noncaseating granulomas. The key to treatment is also immune responses against aggressive endogenous antigens [131]. Currently, there have been no reports of DPSCs in the treatment of CD. However, studies of other MSCs for CD have entered clinical trials and have been shown to be safe and feasible [132,133,134]. Later studies on DPSCs can incorporate CD and provide more options for clinical treatment.

### 5.6. Type 1 Diabetes

Type 1 diabetes (T1D) is a chronic metabolic disease caused by the autoimmune destruction of insulin-secreting pancreatic β-cells, which causes serious chronic complications and irreversible multiple organ damage to patients. These complications include diabetic nephropathy, neuropathy, retinopathy, and cardiovascular disease [135]. T1D is undoubtedly a complex and multifactorial disease involving genetic predisposition and associated with environmental factors that lead to an unbalanced immune response [136]. Immunologically, it is caused by the entry of autoinvasive T lymphocytes into the islets of Langerhans, where they destroy insulin-producing beta cells [137]. Previous studies have demonstrated that most efforts to intervene in the pathogenesis of T1D involve immune-based therapies, with the primary endpoint focused on β-cell function maintenance [138]. DPSCs have been reported to differentiate into β islet-like cells and can be used for autologous stem cell therapy in T1D [139,140,141].

The therapeutic effect of hDPSCs in T1D has been compared in diabetic rats by intravenous (IV) and intrapancreatic (IP) injections [142]. Results revealed that both IV and IP transplantation of DPSCs reduced blood glucose and increased levels of rat and human serum insulin and C-peptide [142]. Expression of the human-specific pancreatic β-cell genes was detected in the pancreatic tissues of both the IP and IV groups, indicating that hDPSCs can migrate and survive in the rat pancreas [142]. Moreover, injected DPSCs exerted pro-angiogenetic and antiapoptotic effects, and promoted endogenous β-cell replication [142]. Datta et al. compared the transplantation of DPSCs via IV or intramuscular (IM) routes by single or two repeated doses in diabetic neuropathy [143]. All treatments were able to down-regulate the expression of TNF-α and IL-6, up-regulate the expression of TGF-β, rapidly improve hyperalgesia, grip strength, motor coordination, and nerve conduction velocity in rats with streptozotocin (STZ)-induced neuropathy, and improve neuropathy caused by T1D [143]. Hata et al. also showed that the treatment effect of a single injection of hDPSCs transplantation lasted a long time and was beneficial for the long-term treatment of diabetic polyneuropathy [144]. IV transplantation of hDPSCs or mouse DPSCs (mDPSCs) also had therapeutic effects on parotid gland injury and renal injury in STZ-induced T1D rats, and also produced powerful and long-lasting anti-nociceptive effects on behavioral neuropathic pain [145,146]. Greene et al. reported that DPSCs contributed to the restoration of a non-healing wound bed by improving cytokines, treating skin wounds, and promoting healing in T1D mice [147]. The in vitro and in vivo study of DPSCs for T1D has entered a very mature stage and can shift the study to clinical trials, providing more possibilities for inclusion in clinical applications.

### 5.7. Psoriasis

Psoriasis is a chronic inflammatory disease of the skin mediated by T cells, dendritic cells, and inflammatory cytokines [148]. Meng et al. confirmed the therapeutic effect of HGF-DPSCs in imiquimod-induced psoriasis [149]. They found that overexpression of HGF could reduce the inflammatory response, enhance the therapeutic effect of DPSCs on psoriasis, and improve the immunoregulatory capacity of DPSCs. By co-culturing with activated PBMCs, it was demonstrated that overexpression of HGF can enhance the immunoregulatory ability of DPSCs by down-regulating Th1 and Th17 cells and up-regulating Treg. In psoriatic mice, both DPSCs and HGF-DPSCs reduce the degree of psoriatic lesions and improve psoriasiform erythema, scaling, and thickening. They also down-regulated cytokeratin 6 and cytokeratin 17 expression. HGF overexpression also promoted the reduction of splenic masses, significantly down-regulated the expression levels of IFN-γ, TNF-α, and IL-17A in serum and T-bet, IFN-γ, retinoid-related orphan receptor-γ (RORγ), IL-17A, IL-17F, and IL-23 in psoriatic lesions, and up-regulated the expression levels of the Treg transcription factor Foxp3 and the Treg-related cytokine IL-10. Although there are few data of DPSCs on this disease, it also provides new evidence for cell therapy in patients with psoriasis.

**Table 3:** In vivo study of dental pulp stem cells in immune-mediated diseases.

Disease	Reference	Animal Type	Injection Type	Dosages and Routes	Duration of the Symptom Improvement	Whether Repeated Injections	Improved Symptoms
Sjögren’s syndrome	Matsuma-ra-Kawashima et al. [113]	NOD mice	DPSC-CM	500 uL, iv	2 weeks	Yes	Reduces inflammation of the submandibular gland and SS-induces hyposalivation
	Ogata et al. [45]	MRL/lpr mice	DPSC-CM	500 uL, iv	2 weeks	Yes	
	Du et al. [111].	NOD mice	SHED	1 × 10^6^, iv	14 weeks	No	
Osteoarthritis	Cui et al. [122]	SD rats	DPSCs	2 × 10^5^, knee joint injections	22 days	No	Improves joint pain, cartilage degradation
	Li et al. [48]	New Zealand White rabbits	DPSCs	1 × 10^6^/2 × 10^5^, knee joint injections	10 weeks	No	
COVID-19	Ye et al. [128]	Adults	DPSCs	3 × 10^7^, iv	4 weeks	Yes	Inhibits the occurrence of inflammation
Ulcerative colitis	Li et al. [50]	SD rats	DPSCs	1 × 10^6^, iv	4 weeks	No	Inhibits inflammatory response, reduces oxidative stress, and alleviates colonic injury
Systemic Lupus Erythematosus	Tang et al. [117]	B6/lpr mice	DPSCs	2 × 10^5^/10 g, iv	10 weeks	No	Reduces hair loss, lymphatics, hepatosplenomegaly, proteinuria, and anti-dsDNA levels
	Makino et al. [30]	MRL/lpr mice	DPSCs	1 × 10^5^/10 g, iv	4 weeks	No	
	Ma et al. [118]	MRL/lpr mice	SHED	1 × 10^5^/10 g, iv	4 weeks	No	
	Sonoda et al. [33]	MRL/lpr mice	SHED	1 × 10^5^/10 g, iv	4 weeks	No	
Psoriasis	Meng et al. [149]	BALB/C mice	DPSCs	2 × 10^6^, iv	6 days	No	Reduces the extent of skin lesions in psoriasis, improves psoriasiform erythema, scaling, and thickening, and promotes the reduction of splenic masses
Type 1 diabetes	EI-Kersh et al. [142]	SD rats	DPSCs	1 × 10^6^, iv or ip	4 weeks	No	Improves hyperalgesia, grip strength, motor coordination, and nerve conduction velocity
	Datta et al. [143]	Wistar rats	DPSCs	1 × 10^6^, iv or im	4 or 8 weeks	All involve	
	Al-Serwi et al. [145]	SD rats	DPSCs	1 × 10^6^, iv	4 weeks	No	
	Guimaraes et al. [146]	C57BL/6 mice	DPSCs	1 × 10^6^, iv	80 days	No	
	Hata et al. [144]	Nude mice	DPSCs	1 × 10^5^, im	16 weeks	No	

NOD: non-obese diabetic; DPSCs: dental pulp stem cells; DPSC-CM: dental pulp stem cells-derived culture medium; SHED: pulp stem cells from exfoliated deciduous teeth; IM: intramuscular; IP: intrapancreatic; IV: intravenous; SS: Sjögren’s syndrome.

## 6. Clinical Perspectives

To date, we have found 11 entries in the www.ClinicalTrials.gov (accessed on 25 April 2023) database on the use of dental-derived mesenchymal stem cells in various diseases. The results of clinical studies have demonstrated the feasibility and safety of DPSCs therapy. Clinical studies have shown that 5 × 10^6^ DPSCs seeded on scaffolds can be used to treat patients with periodontal defects using tissue engineering. Regenerative periodontal treatment with allogeneic DPSCs provided no evidence of immune rejection, and it helped to reduce periodontal pockets and drive the formation of osteoid tissue to actively repair periodontal disease-induced intraosseous defects [150]. Injection of 2.3 × 10^9^ particles/mL SHED into the penis of patients with erectile dysfunction had a favorable effect in patients with erectile dysfunction due to mild to moderate vascular disease [151]. In addition, the safety and efficacy of DPSCs for the treatment of patients with severe COVID-19 was also evaluated [128]. However, due to the narrow age range of subjects, there is insufficient evidence to assess the effect of DPSCs on alveolar healing after third molar extraction [152]. Unfortunately, the clinical applications of DPSCs for the treatment of immune-mediated diseases are currently lacking. However, the efficacy and safety of MSCs in immune-mediated diseases have been evaluated (Table 4). For future clinical work, we need to determine the number of cells, time window, course of treatment, long-term efficacy, and the selection of autologous or allogeneic DPSCs for the treatment of immune diseases. It should be noted that the ability of DPSCs to proliferate and differentiate decreases with an increase in the number of passages, and the immunomodulatory function weakens, so that DPSCs can not be expanded in large quantities at will. Studies have shown that the third generation of cells is proven to be suitable for clinical treatment [16]. At the same time, the inclusion of a sufficient number of subjects is also necessary for a clinical trial.

## 7. Conclusions

As the strong immunomodulatory capacity of stem cells has been discovered, researchers have turned the focus of immune-mediated disease treatment to cell therapy [185,186]. Currently, there are many clinical trials and animal experiments that have evaluated MSCs from different tissue sources for the treatment of immune-mediated diseases, which also includes DPSCs. In terms of immune-mediated diseases, DPSCs are superior to MSCs from other sources for the following reasons: (1) DPSCs are isolated from discarded teeth by less invasive methods and have no ethical considerations; (2) DPSCs are abundant, can be collected multiple times in a person’s life, and have effective therapeutic effects; (3) DPSCs have a higher proliferative capacity, play an important role in tissue regeneration, and have a higher regenerative potential compared with other MSCs; and (4) DPSCs have similar or even better immunomodulatory effects than MSCs from other sources [49]. DPSCs and their exosomes can exert immunomodulatory abilities by acting on target immune cells to regulate cytokines. DPSCs can also migrate to the lesion site to differentiate into target cells to repair the injured tissue. However, studies have shown that MSCs may be transformed in response to cytokines in the inflammatory environment, and thus lose their immunomodulatory ability [187]. The effect of an inflammatory microenvironment on MSCs in autoimmune diseases needs further study. In recent years, many studies have confirmed their great therapeutic potential in immune-mediated diseases, but almost all the current research work on DPSCs are conducted in animal models, with few clinical trials. Therefore, the focus of future work should be on clinical experiments to further test their application in the human body.

**Figure 2:**
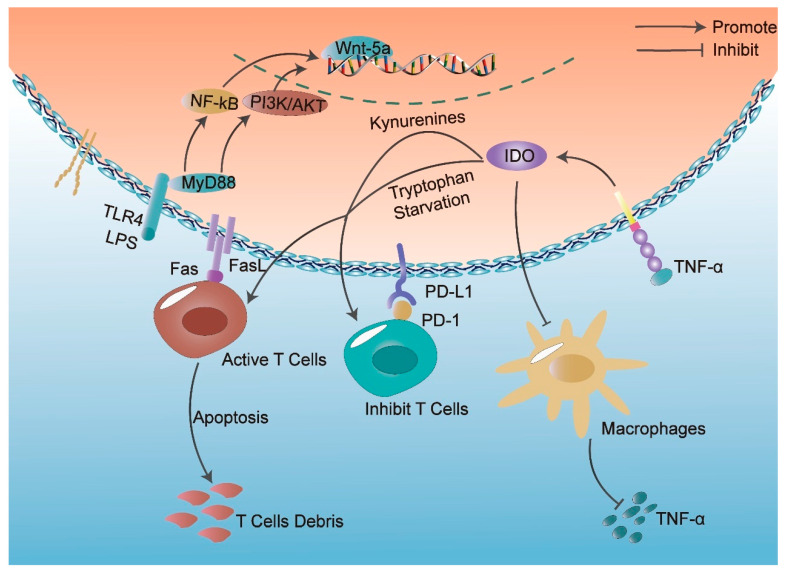
Dental pulp stem cells (DPSCs) exert immunosuppressive effects by mediating different pathways. DPSCs inhibit lipopolysaccharide (LPS)-triggered macrophage secretion of tumor necrosis factor-α (TNF-α) through an indoleamine-2,3-dioxygenase (IDO)-dependent mechanism, and suppress allogeneic T lymphocyte activation through Fas/FasL interaction and induction of apoptosis in Tregs. Programmed cell death (PD)-L1 on DPSCs binds to the receptor PD-1 on activated T cells and suppresses immune responses by counteracting T cell activation signals. When DPSCs were exposed to LPS, DPSCs showed enhanced Toll-like receptors (TLR) 4 expression and also promoted Wnt5a expression through the TLR4/MyD88/PI3-kinase/AKT pathway.

In this review, we discussed the immunomodulatory function mediated by IDO, Fas/FasL, PD1/PDL1, and TLR4, respectively, in terms of the mechanism of immunomodulation by DPSCs (Figure 2). In addition, HGF, NO, and HLA-G [42,50,149] are also involved in the immunoregulatory function of DPSCs, but they are not deeply studied. At current stage, further research on the immunoregulatory mechanism of DPSCs is still needed in order to facilitate the further implementation of clinical research and to provide better ways to control and optimize immune responses. Furthermore, the selection of optimal MSCs for symptomatic treatment of various immune-mediated diseases is also important for future studies. Based on the differentiation ability of DPSCs, how to guide the target cells after differentiation to target and home to the affected organs or to localize the injected DPSCs to the affected organs should also be the direction of future research.

## Figures and Tables

**Table 1:** Effects of soluble factors secreted by DPSCs on target cells.

Soluble Factors	Target Cells	Mediated Effects	References
TGF-β1, IL-6, IL-10, IL-29, IL-13, HLA-G, IDO, ICAM-1, VCAM-1, HGF, VEGF, NO, PGE2	Lymphocyte	Inhibit lymphocyte proliferation	[42,43,45]
TGF-β1, IL-29, TNF-α, HGF, IDO	Macrophage	Inhibit macrophage function	[18,45,48]
IL-6, IL-10, IL-13, M-CSF, IGF-1, GM-CSF, PGE2	Macrophage	Induce m1 differentiation into m2	[45,46,49]
HGF	Hepatocytes	Promotes hepatocyte regeneration	[50]
SCF, VEGF	Endothelial cells	Promote neo-vascularization	[46,51]

TGF-β1: transforming growth factor beta 1; IL: interleukin; HLA-G: human leukocyte antigen; IDO: indoleamine-2,3-dioxygenase; ICAM-1: intercellular cell adhesion molecule; VCAM-1: vascular cell adhesion molecule; HGF: hepatocyte growth factor; VEGF: vascular endothelial growth factor; NO, nitric oxide; PGE2: prostaglandin E2; TNF-α: tumor necrosis factor; M-CSF: macrophage-colony stimulating factor; IGF-1: insulin growth factor-1; GM-CSF: granulocyte macrophage colony-stimulating factor; SCF: stem cell factor.

**Table 2:** Soluble factors that regulate macrophage polarization.

Type of Macrophages	Soluble Factor
M→M1	LPS, IFN-γ, TNF-α, IL-1β
M1→M2	IL-4, IL-13, IL-10, M-CSF, MCP-1, ED-Siglec-9

LPS: lipopolysaccharide; IFN-γ: interferon-r; IL: interleukin; TNF-α: tumor necrosis factor; M-CSF: macrophage-colony stimulating factor MCP-1: monocyte chemoattractant protein-1; ED-Siglec-9: sectodomain of sialic acid-binding Ig-like lectin-9.

**Table 4:** Clinical studies of mesenchymal stem cells in immune-mediated diseases.

Disease	Source	Serious Adverse Events	Conclusion	NCT Number	Reference
Type 1 Diabetes	UCMSC	No	Increased insulin secretion	NCT01374854	[153]
	BMSC	No	Increased C-peptide responsiveness to MMTT	NCT01068951	[154]
	BMSC	No	Improved blood sugar and immune indicators	NCT04078308	[135]
	UCMSC	No	Increased fasting and postprandial C-peptide levels	N/A	[155]
	UCMSC/BMSC	No	Reduced the incidence of diabetes complications	NCT01374854	[156]
	ADMSC/BMSC	No	Effective control of high blood sugar	N/A	[157]
	WJMSC	No	Restored islet beta cell function	N/A	[158]
Osteoarthritis	BMSC	No	Reduced synovial inflammation and cartilage degradation	NCT02351011	[159]
	ADMSC	No	Significant improvement in pain and cartilage quality	NCT01585857	[160]
				NCT02162693	[161]
				NCT02658344	[162]
	UCMSC	No	Significant improvement in pain and cartilage quality	NCT02580695	[163]
	PMSC	No	Significant improvement in pain and cartilage quality	N/A	[164]
	BMSC	No	Significant improvement in pain and cartilage quality	N/A	[165]
				NCT01586312	[166]
				NCT02123368	[167]
				N/A	[168]
Psoriasis	UCMSC	No	Increased Tregs, decreased TH17	NCT03765957	[169]
Systemic Lupus Erythematosus	BMSC	No	Improved disease activity and stabilizes renal function	NCT00698191	[170]
				N/A	[171]
	UCMSC	A patient died of severe pneumonia	Not demonstrate a positive treatment effect.	NCT01539902	[172]
	UCMSC	No	Improved systemic performance of the hematopoietic and dermal systems	NCT01741857	[173]
	BMSC/UCMSC	No	Improved renal function	N/A	[174]
				N/A	[175]
	ADMSC	No	Reduced urinary protein excretion and disease activity	N/A	[176]
Ulcerative Colitis	BMSC	No	Improvement in symptoms with reduced urgency and blood in the stool	NCT04543994	[177]
	BMSC	No	Reduced inflammatory activity and stimulates intestinal mucosal repair	N/A	[178]
COVID-19	UCMSC	No	Patient recovered and discharged from hospital	NCT04288102	[179]
	UCMSC	No	Improvement of lung lesions	NCT04288102	[180]
	BMSC	No	Improved breathing difficulties	NCT04445454	[181]
	MBMSC	No	Improved breathing difficulties	N/A	[182]
	WJMSC	No	Reduced inflammation	N/A	[183]
Periodontitis	UCMSC	No	Reduced sensitivity to dental pain	NCT03102879	[184]
	MSC	No	N/A	NCT04446897	N/A
	ADMSC	No	N/A	NCT04270006	N/A
	DPSC	No	Periodontal tissue regeneration	N/A	[150]
	GMSC	No	N/A	NCT03137979	N/A
	DPSC	No	N/A	NCT04983225	N/A
	PLMSC	No	N/A	NCT01082822	N/A
	BMSC	No	N/A	NCT02449005	N/A

N/A: not applicable; UCMSC: umbilical cord mesenchymal stem cells; ADMSC: adipose mesenchymal stem cells; BMSC: bone marrow mesenchymal stem cells; PMSC: placental mesenchymal stem cells; WJMSC: Wharton’s jelly mesenchymal stem cells; MBMSC: menstrual blood mesenchymal stem cells; PLMSC: periodontal ligament mesenchymal stem cells, GMSC: gingiva mesenchymal stem cells; DPSC: dental pulp stem cell.

## Abbreviations

DPSCs, dental pulp stem cells; MSCs, mesenchymal stem cells; hDPSCs, human dental pulp stem cells; CD, cluster of differentiation; HLA, human leukocyte antigen; BMSCs, bone marrow mesenchymal stem cells; NK, natural killer; DPSC-exo, dental pulp stem cells-derived exosomes; DPSC-EV, extracellular vesicles of dental pulp stem cells; TGF-β, transforming growth factor beta; PBMCs, peripheral blood mononuclear cells; PHA, phytohemagglutinin; Tregs, regulatory T cells; LPS, lipopolysaccharide; SHED-CM, culture medium derived from stem cells from human exfoliated deciduous teeth; SHED, pulp stem cells from exfoliated deciduous teeth; MCP-1, monocyte chemoattractant protein-1; ED-Siglec-9, sectodomain of sialic acid-binding Ig-like lectin-9; ICAM-1, intercellular cell adhesion molecule; VCAM-1, vascular cell adhesion molecule; IGF-1, insulin growth factor-1; DPSC-CM, dental pulp stem cells-derived culture medium; TLR, Toll-like receptors; IDO, indoleamine-2,3-dioxygenase; FasL, Fas ligand; PGE2, prostaglandin E2; NO, nitric oxide; iNOS, inducible nitric oxide synthase; SCF, stem cell factor; VEGF, vascular endothelial growth factor; HGF, hepatocyte growth factor; SS, Sjögren’s syndrome; SLE, systemic lupus erythematosus; SHED-EVs, SHED-releasing extracellular vesicles; ConA, concanavalin A; GM-CSF, granulocyte macrophage colony-stimulating factor; UC, ulcerative colitis; IBD, inflammatory bowel disease; HGF-DPSCs, DPSCs overexpressing HGF; T1D, type 1 diabetes; IV, intravenous; IP, intrapancreatic; IM, intramuscular; STZ, streptozotocin; DCs, dendritic cells; MHC, Major histocompatibility complex; IL, interleukin; M-CSF, macrophage-colony stimulating factor; TNF-α, tumor necrosis factor-α; FADD, Fas-associated death domain; DISC, death-inducing signaling complex; PD-1, Programmed cell death-1; CD, Crohn’s disease; ATP, adenosine triphosphate; ADO, adenosine; IFN-γ, interferon-γ; NOD, non-obese diabetic.

## References

[1] Li N., Hua J. (2017). Interactions between mesenchymal stem cells and the immune system. Cell. Mol. Life Sci..

[2] Han Y., Yang J., Fang J., Zhou Y., Candi E., Wang J., Hua D., Shao C., Shi Y. (2022). The secretion profile of mesenchymal stem cells and potential applications in treating human diseases. Signal Transduct. Target. Ther..

[3] Corselli M., Chen C.W., Crisan M., Lazzari L., Péault B. (2010). Perivascular ancestors of adult multipotent stem cells. Arterioscler. Thromb. Vasc. Biol..

[4] Yianni V., Sharpe P.T. (2019). Perivascular-Derived Mesenchymal Stem Cells. J. Dent. Res..

[5] Huang X.P., Sun Z., Miyagi Y., McDonald Kinkaid H., Zhang L., Weisel R.D., Li R.K. (2010). Differentiation of allogeneic mesenchymal stem cells induces immunogenicity and limits their long-term benefits for myocardial repair. Circulation.

[6] Huang B., Cheng X., Wang H., Huang W., la Ga Hu Z., Wang D., Zhang K., Zhang H., Xue Z., Da Y. (2016). Mesenchymal stem cells and their secreted molecules predominantly ameliorate fulminant hepatic failure and chronic liver fibrosis in mice respectively. J. Transl. Med..

[7] Gupta P.K., Das A.K., Chullikana A., Majumdar A.S. (2012). Mesenchymal stem cells for cartilage repair in osteoarthritis. Stem Cell Res. Ther..

[8] Guillamat-Prats R. (2021). The Role of MSC in Wound Healing, Scarring and Regeneration. Cells.

[9] Chu K.A., Wang S.Y., Yeh C.C., Fu T.W., Fu Y.Y., Ko T.L., Chiu M.M., Chen T.H., Tsai P.J., Fu Y.S. (2019). Reversal of bleomycin-induced rat pulmonary fibrosis by a xenograft of human umbilical mesenchymal stem cells from Wharton’s jelly. Theranostics.

[10] Dabrowska S., Andrzejewska A., Janowski M., Lukomska B. (2020). Immunomodulatory and Regenerative Effects of Mesenchymal Stem Cells and Extracellular Vesicles: Therapeutic Outlook for Inflammatory and Degenerative Diseases. Front. Immunol..

[11] Schiess M., Suescun J., Doursout M.F., Adams C., Green C., Saltarrelli J.G., Savitz S., Ellmore T.M. (2021). Allogeneic Bone Marrow-Derived Mesenchymal Stem Cell Safety in Idiopathic Parkinson’s Disease. Mov. Disord..

[12] Gronthos S., Mankani M., Brahim J., Robey P.G., Shi S. (2000). Postnatal human dental pulp stem cells (DPSCs) in vitro and in vivo. Proc. Natl. Acad. Sci. USA.

[13] Nuti N., Corallo C., Chan B.M., Ferrari M., Gerami-Naini B. (2016). Multipotent Differentiation of Human Dental Pulp Stem Cells: A Literature Review. Stem Cell Rev. Rep..

[14] Al Madhoun A., Sindhu S., Haddad D., Atari M., Ahmad R., Al-Mulla F. (2021). Dental Pulp Stem Cells Derived From Adult Human Third Molar Tooth: A Brief Review. Front. Cell Dev. Biol..

[15] Chen Y.K., Huang A.H., Chan A.W., Shieh T.Y., Lin L.M. (2011). Human dental pulp stem cells derived from different cryopreservation methods of human dental pulp tissues of diseased teeth. J. Oral. Pathol. Med..

[16] Yan M., Nada O.A., Kluwe L., Gosau M., Smeets R., Friedrich R.E. (2020). Expansion of Human Dental Pulp Cells In Vitro Under Different Cryopreservation Conditions. In Vivo.

[17] Alkhayal Z., Shinwari Z., Gaafar A., Alaiya A. (2020). Proteomic Profiling of the First Human Dental Pulp Mesenchymal Stem/Stromal Cells from Carbonic Anhydrase II Deficiency Osteopetrosis Patients. Int. J. Mol. Sci..

[18] Lee S., Zhang Q.Z., Karabucak B., Le A.D. (2016). DPSCs from Inflamed Pulp Modulate Macrophage Function via the TNF-α/IDO Axis. J. Dent. Res..

[19] Sun H.H., Chen B., Zhu Q.L., Kong H., Li Q.H., Gao L.N., Xiao M., Chen F.M., Yu Q. (2014). Investigation of dental pulp stem cells isolated from discarded human teeth extracted due to aggressive periodontitis. Biomaterials.

[20] Kawashima N. (2012). Characterisation of dental pulp stem cells: A new horizon for tissue regeneration?. Arch. Oral. Biol..

[21] Ozdemir A.T., Ozdemir R.B.O., Kirmaz C., Sariboyaci A.E., Halbutogllari Z.S.U., Ozel C., Karaoz E. (2016). The paracrine immunomodulatory interactions between the human dental pulp derived mesenchymal stem cells and CD4 T cell subsets. Cell. Immunol..

[22] Andrukhov O., Behm C., Blufstein A., Rausch-Fan X. (2019). Immunomodulatory properties of dental tissue-derived mesenchymal stem cells: Implication in disease and tissue regeneration. World J. Stem Cells.

[23] Abu Kasim N.H., Govindasamy V., Gnanasegaran N., Musa S., Pradeep P.J., Srijaya T.C., Aziz Z.A. (2015). Unique molecular signatures influencing the biological function and fate of post-natal stem cells isolated from different sources. J. Tissue Eng. Regen. Med..

[24] Nagpal A., Kremer K.L., Hamilton-Bruce M.A., Kaidonis X., Milton A.G., Levi C., Shi S., Carey L., Hillier S., Rose M. (2016). TOOTH (The Open study Of dental pulp stem cell Therapy in Humans): Study protocol for evaluating safety and feasibility of autologous human adult dental pulp stem cell therapy in patients with chronic disability after stroke. Int. J. Stroke.

[25] Carballido J.M., Santamaria P. (2019). Taming autoimmunity: Translating antigen-specific approaches to induce immune tolerance. J. Exp. Med..

[26] Montaño J., Garnica J., Santamaria P. (2021). Immunomodulatory and immunoregulatory nanomedicines for autoimmunity. Semin. Immunol..

[27] Serra P., Santamaria P. (2019). Antigen-specific therapeutic approaches for autoimmunity. Nat. Biotechnol..

[28] Martinez V.G., Ontoria-Oviedo I., Ricardo C.P., Harding S.E., Sacedon R., Varas A., Zapata A., Sepulveda P., Vicente A. (2017). Overexpression of hypoxia-inducible factor 1 alpha improves immunomodulation by dental mesenchymal stem cells. Stem Cell. Res. Ther..

[29] Laing A.G., Fanelli G., Ramirez-Valdez A., Lechler R.I., Lombardi G., Sharpe P.T. (2019). Mesenchymal stem cells inhibit T-cell function through conserved induction of cellular stress. PLoS ONE.

[30] Makino Y., Yamaza H., Akiyama K., Ma L., Hoshino Y., Nonaka K., Terada Y., Kukita T., Shi S., Yamaza T. (2013). Immune therapeutic potential of stem cells from human supernumerary teeth. J. Dent. Res..

[31] de Witte S.F., Franquesa M., Baan C.C., Hoogduijn M.J. (2015). Toward Development of iMesenchymal Stem Cells for Immunomodulatory Therapy. Front. Immunol..

[32] Song N., Scholtemeijer M., Shah K. (2020). Mesenchymal Stem Cell Immunomodulation: Mechanisms and Therapeutic Potential. Trends Pharmacol. Sci..

[33] Sonoda S., Murata S., Kato H., Zakaria F., Kyumoto-Nakamura Y., Uehara N., Yamaza H., Kukita T., Yamaza T. (2021). Targeting of Deciduous Tooth Pulp Stem Cell-Derived Extracellular Vesicles on Telomerase-Mediated Stem Cell Niche and Immune Regulation in Systemic Lupus Erythematosus. J. Immunol..

[34] Wada N., Menicanin D., Shi S., Bartold P.M., Gronthos S. (2009). Immunomodulatory properties of human periodontal ligament stem cells. J. Cell. Physiol..

[35] Liu C., Hu F., Jiao G., Guo Y., Zhou P., Zhang Y., Zhang Z., Yi J., You Y., Li Z. (2022). Dental pulp stem cell-derived exosomes suppress M1 macrophage polarization through the ROS-MAPK-NFκB P65 signaling pathway after spinal cord injury. J. Nanobiotechnol..

[36] Javazon E.H., Beggs K.J., Flake A.W. (2004). Mesenchymal stem cells: Paradoxes of passaging. Exp. Hematol..

[37] Uccelli A., Pistoia V., Moretta L. (2007). Mesenchymal stem cells: A new strategy for immunosuppression?. Trends Immunol..

[38] Ding G., Niu J., Liu Y. (2015). Dental pulp stem cells suppress the proliferation of lymphocytes via transforming growth factor-β1. Hum. Cell.

[39] Tomic S., Djokic J., Vasilijic S., Vucevic D., Todorovic V., Supic G., Colic M. (2011). Immunomodulatory properties of mesenchymal stem cells derived from dental pulp and dental follicle are susceptible to activation by toll-like receptor agonists. Stem Cells Dev..

[40] Kwack K.H., Lee J.M., Park S.H., Lee H.W. (2017). Human Dental Pulp Stem Cells Suppress Alloantigen-induced Immunity by Stimulating T Cells to Release Transforming Growth Factor Beta. J. Endod..

[41] Hong J.W., Lim J.H., Chung C.J., Kang T.J., Kim T.Y., Kim Y.S., Roh T.S., Lew D.H. (2017). Immune Tolerance of Human Dental Pulp-Derived Mesenchymal Stem Cells Mediated by CD4^+^CD25^+^FoxP3^+^ Regulatory T-Cells and Induced by TGF-β1 and IL-10. Yonsei Med. J..

[42] Hossein-Khannazer N., Hashemi S.M., Namaki S., Ghanbarian H., Sattari M., Khojasteh A. (2019). Study of the immunomodulatory effects of osteogenic differentiated human dental pulp stem cells. Life Sci..

[43] Demircan P.C., Sariboyaci A.E., Unal Z.S., Gacar G., Subasi C., Karaoz E. (2011). Immunoregulatory effects of human dental pulp-derived stem cells on T cells: Comparison of transwell co-culture and mixed lymphocyte reaction systems. Cytotherapy.

[44] Matsubara K., Matsushita Y., Sakai K., Kano F., Kondo M., Noda M., Hashimoto N., Imagama S., Ishiguro N., Suzumura A. (2015). Secreted ectodomain of sialic acid-binding Ig-like lectin-9 and monocyte chemoattractant protein-1 promote recovery after rat spinal cord injury by altering macrophage polarity. J. Neurosci..

[45] Ogata K., Matsumura-Kawashima M., Moriyama M., Kawado T., Nakamura S. (2021). Dental pulp-derived stem cell-conditioned media attenuates secondary Sjogren’s syndrome via suppression of inflammatory cytokines in the submandibular glands. Regen. Ther..

[46] Omi M., Hata M., Nakamura N., Miyabe M., Kobayashi Y., Kamiya H., Nakamura J., Ozawa S., Tanaka Y., Takebe J. (2016). Transplantation of dental pulp stem cells suppressed inflammation in sciatic nerves by promoting macrophage polarization towards anti-inflammation phenotypes and ameliorated diabetic polyneuropathy. J. Diabetes Investig..

[47] Wakayama H., Hashimoto N., Matsushita Y., Matsubara K., Yamamoto N., Hasegawa Y., Ueda M., Yamamoto A. (2015). Factors secreted from dental pulp stem cells show multifaceted benefits for treating acute lung injury in mice. Cytotherapy.

[48] Li P.L., Wang Y.X., Zhao Z.D., Li Z.L., Liang J.W., Wang Q., Yin B.F., Hao R.C., Han M.Y., Ding L. (2021). Clinical-grade human dental pulp stem cells suppressed the activation of osteoarthritic macrophages and attenuated cartilaginous damage in a rabbit osteoarthritis model. Stem Cell Res. Ther..

[49] Zayed M., Iohara K. (2020). Immunomodulation and Regeneration Properties of Dental Pulp Stem Cells: A Potential Therapy to Treat Coronavirus Disease 2019. Cell Transplant..

[50] Li N., Zhang Y., Nepal N., Li G., Yang N., Chen H., Lin Q., Ji X., Zhang S., Jin S. (2021). Dental pulp stem cells overexpressing hepatocyte growth factor facilitate the repair of DSS-induced ulcerative colitis. Stem Cell Res. Ther..

[51] Mu X., Shi L., Pan S., He L., Niu Y., Wang X. (2020). A Customized Self-Assembling Peptide Hydrogel-Wrapped Stem Cell Factor Targeting Pulp Regeneration Rich in Vascular-Like Structures. ACS Omega.

[52] Tas S.W., Vervoordeldonk M.J., Hajji N., Schuitemaker J.H., van der Sluijs K.F., May M.J., Ghosh S., Kapsenberg M.L., Tak P.P., de Jong E.C. (2007). Noncanonical NF-kappaB signaling in dendritic cells is required for indoleamine 2,3-dioxygenase (IDO) induction and immune regulation. Blood.

[53] Riccio M., Carnevale G., Cardinale V., Gibellini L., De Biasi S., Pisciotta A., Carpino G., Gentile R., Berloco P.B., Brunelli R. (2014). The Fas/Fas ligand apoptosis pathway underlies immunomodulatory properties of human biliary tree stem/progenitor cells. J. Hepatol..

[54] Pisciotta A., Bertani G., Bertoni L., Di Tinco R., De Biasi S., Vallarola A., Pignatti E., Tupler R., Salvarani C., de Pol A. (2020). Modulation of Cell Death and Promotion of Chondrogenic Differentiation by Fas/FasL in Human Dental Pulp Stem Cells (hDPSCs). Front. Cell Dev. Biol..

[55] Akiyama K., Chen C., Wang D., Xu X., Qu C., Yamaza T., Cai T., Chen W., Sun L., Shi S. (2012). Mesenchymal-stem-cell-induced immunoregulation involves FAS-ligand-/FAS-mediated T cell apoptosis. Cell Stem Cell.

[56] Zhao Y., Wang L., Jin Y., Shi S. (2012). Fas ligand regulates the immunomodulatory properties of dental pulp stem cells. J. Dent. Res..

[57] Genç D., Günaydın B., Sezgin S., Aladağ A., Tarhan E.F. (2022). Immunoregulatory effects of dental mesenchymal stem cells on T and B lymphocyte responses in primary Sjögren’s syndrome. Immunotherapy.

[58] Sharpe A.H., Pauken K.E. (2018). The diverse functions of the PD1 inhibitory pathway. Nat. Rev. Immunol..

[59] Sun C., Mezzadra R., Schumacher T.N. (2018). Regulation and Function of the PD-L1 Checkpoint. Immunity.

[60] Liu Y., Jing H., Kou X., Chen C., Liu D., Jin Y., Lu L., Shi S. (2018). PD-1 is required to maintain stem cell properties in human dental pulp stem cells. Cell Death Differ..

[61] Di Tinco R., Bertani G., Pisciotta A., Bertoni L., Pignatti E., Maccaferri M., Bertacchini J., Sena P., Vallarola A., Tupler R. (2021). Role of PD-L1 in licensing immunoregulatory function of dental pulp mesenchymal stem cells. Stem Cell Res. Ther..

[62] Pignatti E., Pisciotta A., Bertani G., Di Tinco R., Bertoni L., Croci S., Bonacini M., Azzoni P., De Pol A., Salvarani C. (2020). Role of mesenchymal stem cells isolated from dental pulp (DPSCs) in immunoregulation processes mediated by programmed death-ligand 1 (PD-L1). Ann. Rheum. Dis..

[63] Tripathi S., Guleria I. (2015). Role of PD1/PDL1 pathway, and TH17 and treg cells in maternal tolerance to the fetus. Biomed. J..

[64] Germanidis G., Argentou N., Hytiroglou P., Vassiliadis T., Patsiaoura K., Germenis A.E., Speletas M. (2013). Liver FOXP3 and PD1/PDL1 Expression is Down-Regulated in Chronic HBV Hepatitis on Maintained Remission Related to the Degree of Inflammation. Front. Immunol..

[65] Yogev N., Frommer F., Lukas D., Kautz-Neu K., Karram K., Ielo D., von Stebut E., Probst H.C., van den Broek M., Riethmacher D. (2012). Dendritic cells ameliorate autoimmunity in the CNS by controlling the homeostasis of PD-1 receptor(+) regulatory T cells. Immunity.

[66] Liu Y., Gao Y., Zhan X., Cui L., Xu S., Ma D., Yue J., Wu B., Gao J. (2014). TLR4 activation by lipopolysaccharide and Streptococcus mutans induces differential regulation of proliferation and migration in human dental pulp stem cells. J. Endod..

[67] He W., Qu T., Yu Q., Wang Z., Lv H., Zhang J., Zhao X., Wang P. (2013). LPS induces IL-8 expression through TLR4, MyD88, NF-kappaB and MAPK pathways in human dental pulp stem cells. Int. Endod. J..

[68] He W., Wang Z., Zhou Z., Zhang Y., Zhu Q., Wei K., Lin Y., Cooper P.R., Smith A.J., Yu Q. (2014). Lipopolysaccharide enhances Wnt5a expression through toll-like receptor 4, myeloid differentiating factor 88, phosphatidylinositol 3-OH kinase/AKT and nuclear factor kappa B pathways in human dental pulp stem cells. J. Endod..

[69] Li S., Luo L., He Y., Li R., Xiang Y., Xing Z., Li Y., Albashari A.A., Liao X., Zhang K. (2021). Dental pulp stem cell-derived exosomes alleviate cerebral ischaemia-reperfusion injury through suppressing inflammatory response. Cell Prolif..

[70] Mancuso P., Raman S., Glynn A., Barry F., Murphy J.M. (2019). Mesenchymal Stem Cell Therapy for Osteoarthritis: The Critical Role of the Cell Secretome. Front. Bioeng. Biotechnol..

[71] Estúa-Acosta G.A., Buentello-Volante B., Magaña-Guerrero F.S., Flores J.E., Vivanco-Rojas O., Castro-Salas I., Zarco-Ávila K., García-Mejía M.A., Garfias Y. (2022). Human Amniotic Membrane Mesenchymal Stem Cell-Synthesized PGE(2) Exerts an Immunomodulatory Effect on Neutrophil Extracellular Trap in a PAD-4-Dependent Pathway through EP2 and EP4. Cells.

[72] Braun D., Longman R.S., Albert M.L. (2005). A two-step induction of indoleamine 2,3 dioxygenase (IDO) activity during dendritic-cell maturation. Blood.

[73] Bouffi C., Bony C., Courties G., Jorgensen C., Noël D. (2010). IL-6-dependent PGE2 secretion by mesenchymal stem cells inhibits local inflammation in experimental arthritis. PLoS ONE.

[74] Kumar B.V., Connors T.J., Farber D.L. (2018). Human T Cell Development, Localization, and Function throughout Life. Immunity.

[75] Yazid F.B., Gnanasegaran N., Kunasekaran W., Govindasamy V., Musa S. (2014). Comparison of immunodulatory properties of dental pulp stem cells derived from healthy and inflamed teeth. Clin. Oral. Investig..

[76] Pierdomenico L., Bonsi L., Calvitti M., Rondelli D., Arpinati M., Chirumbolo G., Becchetti E., Marchionni C., Alviano F., Fossati V. (2005). Multipotent mesenchymal stem cells with immunosuppressive activity can be easily isolated from dental pulp. Transplantation.

[77] Miyara M., Ito Y., Sakaguchi S. (2014). TREG-cell therapies for autoimmune rheumatic diseases. Nat. Rev. Rheumatol..

[78] Swain S.L., Agrewala J.N., Brown D.M., Jelley-Gibbs D.M., Golech S., Huston G., Jones S.C., Kamperschroer C., Lee W.H., McKinstry K.K. (2006). CD4+ T-cell memory: Generation and multi-faceted roles for CD4+ T cells in protective immunity to influenza. Immunol. Rev..

[79] Ji L., Bao L., Gu Z., Zhou Q., Liang Y., Zheng Y., Xu Y., Zhang X., Feng X. (2019). Comparison of immunomodulatory properties of exosomes derived from bone marrow mesenchymal stem cells and dental pulp stem cells. Immunol. Res..

[80] Zhang Z., Ji J., Dong C., Gu Z. (2020). Mir-21 in exosomes drived from dental pulp stem cells ameliorate the tregs/th17 immune response via targeting stat3 in collagen-induced arthritis mice. Ann. Rheum. Dis..

[81] Corcione A., Benvenuto F., Ferretti E., Giunti D., Cappiello V., Cazzanti F., Risso M., Gualandi F., Mancardi G.L., Pistoia V. (2006). Human mesenchymal stem cells modulate B-cell functions. Blood.

[82] Rasmusson I., Le Blanc K., Sundberg B., Ringdén O. (2007). Mesenchymal stem cells stimulate antibody secretion in human B cells. Scand. J. Immunol..

[83] Asari S., Itakura S., Ferreri K., Liu C.P., Kuroda Y., Kandeel F., Mullen Y. (2009). Mesenchymal stem cells suppress B-cell terminal differentiation. Exp. Hematol..

[84] Tabera S., Pérez-Simón J.A., Díez-Campelo M., Sánchez-Abarca L.I., Blanco B., López A., Benito A., Ocio E., Sánchez-Guijo F.M., Cañizo C. (2008). The effect of mesenchymal stem cells on the viability, proliferation and differentiation of B-lymphocytes. Haematologica.

[85] Moretta A., Bottino C., Vitale M., Pende D., Biassoni R., Mingari M.C., Moretta L. (1996). Receptors for HLA class-I molecules in human natural killer cells. Annu. Rev. Immunol..

[86] Yan F., Liu O., Zhang H., Zhou Y., Zhou D., Zhou Z., He Y., Tang Z., Wang S. (2019). Human dental pulp stem cells regulate allogeneic NK cells’ function via induction of anti-inflammatory purinergic signalling in activated NK cells. Cell Prolif..

[87] Najar M., Fayyad-Kazan M., Meuleman N., Bron D., Fayyad-Kazan H., Lagneaux L. (2018). Mesenchymal stromal cells of the bone marrow and natural killer cells: Cell interactions and cross modulation. J. Cell Commun. Signal..

[88] Najar M., Fayyad-Kazan M., Merimi M., Meuleman N., Bron D., Fayyad-Kazan H., Lagneaux L. (2019). Reciprocal immuno-biological alterations occur during the co-culture of natural killer cells and adipose tissue-derived mesenchymal stromal cells. Cytotechnology.

[89] Najar M., Fayyad-Kazan M., Meuleman N., Bron D., Fayyad-Kazan H., Lagneaux L. (2018). Immunological impact of Wharton’s Jelly mesenchymal stromal cells and natural killer cell co-culture. Mol. Cell. Biochem..

[90] Najar M., Fayyad-Kazan M., Meuleman N., Bron D., Fayyad-Kazan H., Lagneaux L. (2018). Immunomodulatory effects of foreskin mesenchymal stromal cells on natural killer cells. J. Cell. Physiol..

[91] Spaggiari G.M., Capobianco A., Abdelrazik H., Becchetti F., Mingari M.C., Moretta L. (2008). Mesenchymal stem cells inhibit natural killer-cell proliferation, cytotoxicity, and cytokine production: Role of indoleamine 2,3-dioxygenase and prostaglandin E2. Blood.

[92] Abbasi B., Shamsasenjan K., Ahmadi M., Beheshti S.A., Saleh M. (2022). Mesenchymal stem cells and natural killer cells interaction mechanisms and potential clinical applications. Stem Cell Res. Ther..

[93] Spaggiari G.M., Capobianco A., Becchetti S., Mingari M.C., Moretta L. (2006). Mesenchymal stem cell-natural killer cell interactions: Evidence that activated NK cells are capable of killing MSCs, whereas MSCs can inhibit IL-2-induced NK-cell proliferation. Blood.

[94] Jewett A., Arasteh A., Tseng H.C., Behel A., Arasteh H., Yang W., Cacalano N.A., Paranjpe A. (2010). Strategies to rescue mesenchymal stem cells (MSCs) and dental pulp stem cells (DPSCs) from NK cell mediated cytotoxicity. PLoS ONE.

[95] Murray P.J., Wynn T.A. (2011). Protective and pathogenic functions of macrophage subsets. Nat. Rev. Immunol..

[96] Lumeng C.N., Bodzin J.L., Saltiel A.R. (2007). Obesity induces a phenotypic switch in adipose tissue macrophage polarization. J. Clin. Investig..

[97] Zheng J., Kong Y., Hu X., Li Z., Li Y., Zhong Y., Wei X., Ling J. (2020). MicroRNA-enriched small extracellular vesicles possess odonto-immunomodulatory properties for modulating the immune response of macrophages and promoting odontogenesis. Stem Cell Res. Ther..

[98] Kano F., Matsubara K., Ueda M., Hibi H., Yamamoto A. (2017). Secreted Ectodomain of Sialic Acid-Binding Ig-Like Lectin-9 and Monocyte Chemoattractant Protein-1 Synergistically Regenerate Transected Rat Peripheral Nerves by Altering Macrophage Polarity. Stem Cells.

[99] Steinman R.M., Nussenzweig M.C. (2002). Avoiding horror autotoxicus: The importance of dendritic cells in peripheral T cell tolerance. Proc. Natl. Acad. Sci. USA.

[100] Chiesa S., Morbelli S., Morando S., Massollo M., Marini C., Bertoni A., Frassoni F., Bartolomé S.T., Sambuceti G., Traggiai E. (2011). Mesenchymal stem cells impair in vivo T-cell priming by dendritic cells. Proc. Natl. Acad. Sci. USA.

[101] Jiang X.X., Zhang Y., Liu B., Zhang S.X., Wu Y., Yu X.D., Mao N. (2005). Human mesenchymal stem cells inhibit differentiation and function of monocyte-derived dendritic cells. Blood.

[102] Aggarwal S., Pittenger M.F. (2005). Human mesenchymal stem cells modulate allogeneic immune cell responses. Blood.

[103] Spaggiari G.M., Abdelrazik H., Becchetti F., Moretta L. (2009). MSCs inhibit monocyte-derived DC maturation and function by selectively interfering with the generation of immature DCs: Central role of MSC-derived prostaglandin E2. Blood.

[104] Fox R.I. (2005). Sjögren’s syndrome. Lancet.

[105] Borchers A.T., Naguwa S.M., Keen C.L., Gershwin M.E. (2003). Immunopathogenesis of Sjögren’s syndrome. Clin. Rev. Allergy Immunol..

[106] Mavragani C.P., Moutsopoulos H.M. (2020). Sjögren’s syndrome: Old and new therapeutic targets. J. Autoimmun..

[107] Del Papa N., Minniti A., Lorini M., Carbonelli V., Maglione W., Pignataro F., Montano N., Caporali R., Vitali C. (2021). The Role of Interferons in the Pathogenesis of Sjögren’s Syndrome and Future Therapeutic Perspectives. Biomolecules.

[108] Abu-Helu R.F., Dimitriou I.D., Kapsogeorgou E.K., Moutsopoulos H.M., Manoussakis M.N. (2001). Induction of salivary gland epithelial cell injury in Sjogren’s syndrome: In vitro assessment of T cell-derived cytokines and Fas protein expression. J. Autoimmun..

[109] Maciejewski J., Selleri C., Anderson S., Young N.S. (1995). Fas antigen expression on CD34+ human marrow cells is induced by interferon gamma and tumor necrosis factor alpha and potentiates cytokine-mediated hematopoietic suppression in vitro. Blood.

[110] Larché M.J. (2006). A short review of the pathogenesis of Sjögren’s syndrome. Autoimmun. Rev..

[111] Du Z.H., Ding C., Zhang Q., Zhang Y., Ge X.Y., Li S.L., Yu G.Y. (2019). Stem cells from exfoliated deciduous teeth alleviate hyposalivation caused by Sjogren syndrome. Oral. Dis..

[112] Caplan A.I., Correa D. (2011). The MSC: An injury drugstore. Cell Stem Cell.

[113] Matsumura-Kawashima M., Ogata K., Moriyama M., Murakami Y., Kawado T., Nakamura S. (2021). Secreted factors from dental pulp stem cells improve Sjögren’s syndrome via regulatory T cell-mediated immunosuppression. Stem Cell Res. Ther..

[114] Kiriakidou M., Ching C.L. (2020). Systemic Lupus Erythematosus. Ann. Intern. Med..

[115] Sun L.Y., Zhang H.Y., Feng X.B., Hou Y.Y., Lu L.W., Fan L.M. (2007). Abnormality of bone marrow-derived mesenchymal stem cells in patients with systemic lupus erythematosus. Lupus.

[116] Sonoda S., Yamaza T. (2022). A New Target of Dental Pulp-Derived Stem Cell-Based Therapy on Recipient Bone Marrow Niche in Systemic Lupus Erythematosus. Int. J. Mol. Sci..

[117] Tang X., Li W., Wen X., Zhang Z., Chen W., Yao G., Chen H., Wang D., Shi S., Sun L. (2019). Transplantation of dental tissue-derived mesenchymal stem cells ameliorates nephritis in lupus mice. Ann. Transl. Med..

[118] Ma L., Makino Y., Yamaza H., Akiyama K., Hoshino Y., Song G., Kukita T., Nonaka K., Shi S., Yamaza T. (2012). Cryopreserved dental pulp tissues of exfoliated deciduous teeth is a feasible stem cell resource for regenerative medicine. PLoS ONE.

[119] Dieppe P.A., Lohmander L.S. (2005). Pathogenesis and management of pain in osteoarthritis. Lancet.

[120] Lamo-Espinosa J.M., Mora G., Blanco J.F., Granero-Moltó F., Núñez-Córdoba J.M., López-Elío S., Andreu E., Sánchez-Guijo F., Aquerreta J.D., Bondía J.M. (2018). Intra-articular injection of two different doses of autologous bone marrow mesenchymal stem cells versus hyaluronic acid in the treatment of knee osteoarthritis: Long-term follow up of a multicenter randomized controlled clinical trial (phase I/II). J. Transl. Med..

[121] Zhang H., Cai D., Bai X. (2020). Macrophages regulate the progression of osteoarthritis. Osteoarthr. Cartil..

[122] Cui S.J., Zhang T., Fu Y., Liu Y., Gan Y.H., Zhou Y.H., Yang R.L., Wang X.D. (2020). Attenuate Experimental Progressive TMJ Arthritis by Inhibiting the STAT1 Pathway. J. Dent. Res..

[123] Lo Monaco M., Gervois P., Beaumont J., Clegg P., Bronckaers A., Vandeweerd J.-M., Lambrichts I. (2020). Therapeutic Potential of Dental Pulp Stem Cells and Leukocyte- and Platelet-Rich Fibrin for Osteoarthritis. Cells.

[124] Lin T., Wu N., Wang L., Zhang R., Pan R., Chen Y.F. (2021). Inhibition of chondrocyte apoptosis in a rat model of osteoarthritis by exosomes derived from miR-140-5p-overexpressing human dental pulp stem cells. Int. J. Mol. Med..

[125] Mata M., Milian L., Oliver M., Zurriaga J., Sancho-Tello M., de Llano J.J.M., Carda C. (2017). In Vivo Articular Cartilage Regeneration Using Human Dental Pulp Stem Cells Cultured in an Alginate Scaffold: A Preliminary Study. Stem Cells Int..

[126] Metcalfe S.M. (2020). Mesenchymal stem cells and management of COVID-19 pneumonia. Med. Drug. Discov..

[127] Croci S., Bonacini M., Dolci G., Massari M., Facciolongo N., Pignatti E., Pisciotta A., Carnevale G., Negro A., Cassone G. (2020). Human Dental Pulp Stem Cells Modulate Cytokine Production in vitro by Peripheral Blood Mononuclear Cells From Coronavirus Disease 2019 Patients. Front. Cell Dev. Biol..

[128] Ye Q., Wang H., Xia X., Zhou C., Liu Z., Xia Z.E., Zhang Z., Zhao Y., Yehenala J., Wang S. (2020). Safety and efficacy assessment of allogeneic human dental pulp stem cells to treat patients with severe COVID-19: Structured summary of a study protocol for a randomized controlled trial (Phase I/II). Trials.

[129] Kumar M., Garand M., Al Khodor S. (2019). Integrating omics for a better understanding of Inflammatory Bowel Disease: A step towards personalized medicine. J. Transl. Med..

[130] Conrad K., Roggenbuck D., Laass M.W. (2014). Diagnosis and classification of ulcerative colitis. Autoimmun. Rev..

[131] Xie M., Qin H., Luo Q., He X., He X., Lan P., Lian L. (2017). Comparison of Adipose-Derived and Bone Marrow Mesenchymal Stromal Cells in a Murine Model of Crohn’s Disease. Dig. Dis. Sci..

[132] Duijvestein M., Vos A.C., Roelofs H., Wildenberg M.E., Wendrich B.B., Verspaget H.W., Kooy-Winkelaar E.M., Koning F., Zwaginga J.J., Fidder H.H. (2010). Autologous bone marrow-derived mesenchymal stromal cell treatment for refractory luminal Crohn’s disease: Results of a phase I study. Gut.

[133] Barnhoorn M.C., Wasser M., Roelofs H., Maljaars P.W.J., Molendijk I., Bonsing B.A., Oosten L.E.M., Dijkstra G., van der Woude C.J., Roelen D.L. (2020). Long-term Evaluation of Allogeneic Bone Marrow-derived Mesenchymal Stromal Cell Therapy for Crohn’s Disease Perianal Fistulas. J. Crohn’s Colitis.

[134] Vieujean S., Loly J.P., Boutaffala L., Meunier P., Reenaers C., Briquet A., Lechanteur C., Baudoux E., Beguin Y., Louis E. (2022). Mesenchymal Stem Cell Injection in Crohn’s Disease Strictures: A Phase I-II Clinical Study. J. Crohn’s Colitis.

[135] Izadi M., Sadr Hashemi Nejad A., Moazenchi M., Masoumi S., Rabbani A., Kompani F., Hedayati Asl A.A., Abbasi Kakroodi F., Jaroughi N., Mohseni Meybodi M.A. (2022). Mesenchymal stem cell transplantation in newly diagnosed type-1 diabetes patients: A phase I/II randomized placebo-controlled clinical trial. Stem Cell Res. Ther..

[136] Morran M.P., Vonberg A., Khadra A., Pietropaolo M. (2015). Immunogenetics of type 1 diabetes mellitus. Mol. Aspects Med..

[137] von Herrath M., Rottembourg D., Bresson D. (2006). Progress in the development of immune-based therapies for type 1 diabetes mellitus. BioDrugs.

[138] von Herrath M., Peakman M., Roep B. (2013). Progress in immune-based therapies for type 1 diabetes. Clin. Exp. Immunol..

[139] Mo Y., Wang Z., Gao J., Yan Y., Ren H., Zhang F., Qi N., Chen Y. (2021). Comparative study of three types of mesenchymal stem cell to differentiate into pancreatic beta-like cells in vitro. Exp. Ther. Med..

[140] Govindasamy V., Ronald V.S., Abdullah A.N., Nathan K.R.G., Aziz Z.A.C.A., Abdullah M., Musa S., Abu Kasim N.H., Bhonde R.R. (2011). Differentiation of Dental Pulp Stem Cells into Islet-like Aggregates. J. Dent. Res..

[141] Xu B., Fan D., Zhao Y., Li J., Wang Z., Wang J., Wang X., Guan Z., Niu B. (2019). Three-Dimensional Culture Promotes the Differentiation of Human Dental Pulp Mesenchymal Stem Cells Into Insulin-Producing Cells for Improving the Diabetes Therapy. Front. Pharmacol..

[142] El-Kersh A., El-Akabawy G., Al-Serwi R.H. (2020). Transplantation of human dental pulp stem cells in streptozotocin-induced diabetic rats. Anat. Sci. Int..

[143] Datta I., Bhadri N., Shahani P., Majumdar D., Sowmithra S., Razdan R., Bhonde R. (2017). Functional recovery upon human dental pulp stem cell transplantation in a diabetic neuropathy rat model. Cytotherapy.

[144] Hata M., Omi M., Kobayashi Y., Nakamura N., Miyabe M., Ito M., Ohno T., Imanishi Y., Himeno T., Kamiya H. (2021). Sustainable Effects of Human Dental Pulp Stem Cell Transplantation on Diabetic Polyneuropathy in Streptozotocine-Induced Type 1 Diabetes Model Mice. Cells.

[145] Al-Serwi R.H., El-Kersh A.O.F.O., El-Akabawy G. (2021). Human dental pulp stem cells attenuate streptozotocin-induced parotid gland injury in rats. Stem Cell Res. Ther..

[146] Guimaraes E.T., Cruz G.d.S., de Almeida T.F., de Freitas Souza B.S., Kaneto C.M., Vasconcelos J.F., Conrado dos Santos W.L., Ribeiro-dos-Santos R., Villarreal C.F., Pereira Soares M.B. (2013). Transplantation of Stem Cells Obtained From Murine Dental Pulp Improves Pancreatic Damage, Renal Function, and Painful Diabetic Neuropathy in Diabetic Type 1 Mouse Model. Cell Transplant..

[147] Greene C., Das H. (2021). Development of Cutaneous Wound in Diabetic Immunocompromised Mice and Use of Dental Pulp-Derived Stem Cell Product for Healing. Methods Mol. Biol..

[148] Bowcock A.M., Krueger J.G. (2005). Getting under the skin: The immunogenetics of psoriasis. Nat. Rev. Immunol..

[149] Meng H., Wei F., Zhou Y., Hu L., Ge Z., Jin J., Wang H., Wu C.T. (2021). Overexpression of Hepatocyte Growth Factor in Dental Pulp Stem Cells Ameliorates the Severity of Psoriasis by Reducing Inflammatory Responses. Stem Cells Dev..

[150] Hernández-Monjaraz B., Santiago-Osorio E., Ledesma-Martínez E., Alcauter-Zavala A., Mendoza-Núñez V.M. (2018). Retrieval of a periodontally compromised tooth by allogeneic grafting of mesenchymal stem cells from dental pulp: A case report. J. Int. Med. Res..

[151] Koga S., Horiguchi Y. (2022). Efficacy of a cultured conditioned medium of exfoliated deciduous dental pulp stem cells in erectile dysfunction patients. J. Cell. Mol. Med..

[152] Barbier L., Ramos E., Mendiola J., Rodriguez O., Santamaria G., Santamaria J., Arteagoitia I. (2018). Autologous dental pulp mesenchymal stem cells for inferior third molar post-extraction socket healing: A split-mouth randomised clinical trial. Med. Oral. Patol. Oral. Cir. Bucal.

[153] Cai J., Wu Z., Xu X., Liao L., Chen J., Huang L., Wu W., Luo F., Wu C., Pugliese A. (2016). Umbilical Cord Mesenchymal Stromal Cell With Autologous Bone Marrow Cell Transplantation in Established Type 1 Diabetes: A Pilot Randomized Controlled Open-Label Clinical Study to Assess Safety and Impact on Insulin Secretion. Diabetes Care.

[154] Carlsson P.O., Schwarcz E., Korsgren O., Le Blanc K. (2015). Preserved β-cell function in type 1 diabetes by mesenchymal stromal cells. Diabetes.

[155] Lu J., Shen S.M., Ling Q., Wang B., Li L.R., Zhang W., Qu D.D., Bi Y., Zhu D.L. (2021). One repeated transplantation of allogeneic umbilical cord mesenchymal stromal cells in type 1 diabetes: An open parallel controlled clinical study. Stem Cell Res. Ther..

[156] Wu Z., Xu X., Cai J., Chen J., Huang L., Wu W., Pugliese A., Li S., Ricordi C., Tan J. (2022). Prevention of chronic diabetic complications in type 1 diabetes by co-transplantation of umbilical cord mesenchymal stromal cells and autologous bone marrow: A pilot randomized controlled open-label clinical study with 8-year follow-up. Cytotherapy.

[157] Thakkar U.G., Trivedi H.L., Vanikar A.V., Dave S.D. (2015). Insulin-secreting adipose-derived mesenchymal stromal cells with bone marrow-derived hematopoietic stem cells from autologous and allogenic sources for type 1 diabetes mellitus. Cytotherapy.

[158] Hu J., Yu X., Wang Z., Wang F., Wang L., Gao H., Chen Y., Zhao W., Jia Z., Yan S. (2013). Long term effects of the implantation of Wharton’s jelly-derived mesenchymal stem cells from the umbilical cord for newly-onset type 1 diabetes mellitus. Endocr. J..

[159] Chahal J., Gómez-Aristizábal A., Shestopaloff K., Bhatt S., Chaboureau A., Fazio A., Chisholm J., Weston A., Chiovitti J., Keating A. (2019). Bone Marrow Mesenchymal Stromal Cell Treatment in Patients with Osteoarthritis Results in Overall Improvement in Pain and Symptoms and Reduces Synovial Inflammation. Stem Cells Transl. Med..

[160] Pers Y.M., Rackwitz L., Ferreira R., Pullig O., Delfour C., Barry F., Sensebe L., Casteilla L., Fleury S., Bourin P. (2016). Adipose Mesenchymal Stromal Cell-Based Therapy for Severe Osteoarthritis of the Knee: A Phase I Dose-Escalation Trial. Stem Cells Transl. Med..

[161] Lu L., Dai C., Zhang Z., Du H., Li S., Ye P., Fu Q., Zhang L., Wu X., Dong Y. (2019). Treatment of knee osteoarthritis with intra-articular injection of autologous adipose-derived mesenchymal progenitor cells: A prospective, randomized, double-blind, active-controlled, phase IIb clinical trial. Stem Cell. Res. Ther..

[162] Lee W.S., Kim H.J., Kim K.I., Kim G.B., Jin W. (2019). Intra-Articular Injection of Autologous Adipose Tissue-Derived Mesenchymal Stem Cells for the Treatment of Knee Osteoarthritis: A Phase IIb, Randomized, Placebo-Controlled Clinical Trial. Stem Cells Transl. Med..

[163] Matas J., Orrego M., Amenabar D., Infante C., Tapia-Limonchi R., Cadiz M.I., Alcayaga-Miranda F., González P.L., Muse E., Khoury M. (2019). Umbilical Cord-Derived Mesenchymal Stromal Cells (MSCs) for Knee Osteoarthritis: Repeated MSC Dosing Is Superior to a Single MSC Dose and to Hyaluronic Acid in a Controlled Randomized Phase I/II Trial. Stem Cells Transl. Med..

[164] Khalifeh Soltani S., Forogh B., Ahmadbeigi N., Hadizadeh Kharazi H., Fallahzadeh K., Kashani L., Karami M., Kheyrollah Y., Vasei M. (2019). Safety and efficacy of allogenic placental mesenchymal stem cells for treating knee osteoarthritis: A pilot study. Cytotherapy.

[165] Emadedin M., Labibzadeh N., Liastani M.G., Karimi A., Jaroughi N., Bolurieh T., Hosseini S.E., Baharvand H., Aghdami N. (2018). Intra-articular implantation of autologous bone marrow-derived mesenchymal stromal cells to treat knee osteoarthritis: A randomized, triple-blind, placebo-controlled phase 1/2 clinical trial. Cytotherapy.

[166] Vega A., Martín-Ferrero M.A., Del Canto F., Alberca M., García V., Munar A., Orozco L., Soler R., Fuertes J.J., Huguet M. (2015). Treatment of Knee Osteoarthritis With Allogeneic Bone Marrow Mesenchymal Stem Cells: A Randomized Controlled Trial. Transplantation.

[167] Lamo-Espinosa J.M., Prósper F., Blanco J.F., Sánchez-Guijo F., Alberca M., García V., González-Vallinas M., García-Sancho J. (2021). Long-term efficacy of autologous bone marrow mesenchymal stromal cells for treatment of knee osteoarthritis. J. Transl. Med..

[168] Orozco L., Munar A., Soler R., Alberca M., Soler F., Huguet M., Sentís J., Sánchez A., García-Sancho J. (2013). Treatment of knee osteoarthritis with autologous mesenchymal stem cells: A pilot study. Transplantation.

[169] Cheng L., Wang S., Peng C., Zou X., Yang C., Mei H., Li C., Su X., Xiao N., Ouyang Q. (2022). Human umbilical cord mesenchymal stem cells for psoriasis: A phase 1/2a, single-arm study. Signal. Transduct. Target. Ther..

[170] Liang J., Zhang H., Hua B., Wang H., Lu L., Shi S., Hou Y., Zeng X., Gilkeson G.S., Sun L. (2010). Allogenic mesenchymal stem cells transplantation in refractory systemic lupus erythematosus: A pilot clinical study. Ann. Rheum. Dis..

[171] Li X., Wang D., Liang J., Zhang H., Sun L. (2013). Mesenchymal SCT ameliorates refractory cytopenia in patients with systemic lupus erythematosus. Bone Marrow Transplant..

[172] Deng D., Zhang P., Guo Y., Lim T.O. (2017). A randomised double-blind, placebo-controlled trial of allogeneic umbilical cord-derived mesenchymal stem cell for lupus nephritis. Ann. Rheum. Dis..

[173] Wang D., Li J., Zhang Y., Zhang M., Chen J., Li X., Hu X., Jiang S., Shi S., Sun L. (2014). Umbilical cord mesenchymal stem cell transplantation in active and refractory systemic lupus erythematosus: A multicenter clinical study. Arthritis Res. Ther..

[174] Gu F., Wang D., Zhang H., Feng X., Gilkeson G.S., Shi S., Sun L. (2014). Allogeneic mesenchymal stem cell transplantation for lupus nephritis patients refractory to conventional therapy. Clin. Rheumatol..

[175] Wang D., Zhang H., Liang J., Li X., Feng X., Wang H., Hua B., Liu B., Lu L., Gilkeson G.S. (2013). Allogeneic mesenchymal stem cell transplantation in severe and refractory systemic lupus erythematosus: 4 years of experience. Cell Transplant..

[176] Ranjbar A., Hassanzadeh H., Jahandoust F., Miri R., Bidkhori H.R., Monzavi S.M., Sanjar-Moussavi N., Matin M.M., Shariati-Sarabi Z. (2022). Allogeneic adipose-derived mesenchymal stromal cell transplantation for refractory lupus nephritis: Results of a phase I clinical trial. Curr. Res. Transl. Med..

[177] Lightner A.L., Dadgar N., Matyas C., Elliott K., Fulmer C., Khaitan N., Ream J., Nachand D., Steele S.R. (2022). A phase IB/IIA study of remestemcel-L, an allogeneic bone marrow-derived mesenchymal stem cell product, for the treatment of medically refractory ulcerative colitis: An interim analysis. Color. Dis..

[178] Lazebnik L.B., Kniazev O.V., Konopliannikov A.G., Parfenov A.I., Ruchkina I.N., Mikhaĭlova Z.F., Tsaregorodtseva T.M., Khomeriki S.G., Rogozina V.A., Gudkova R.B. (2010). [Allogeneic mesenchymal stromal cells in patients with ulcerative colitis: Two years of observation]. Eksp. Klin. Gastroenterol..

[179] Meng F., Xu R., Wang S., Xu Z., Zhang C., Li Y., Yang T., Shi L., Fu J., Jiang T. (2020). Human umbilical cord-derived mesenchymal stem cell therapy in patients with COVID-19: A phase 1 clinical trial. Signal. Transduct. Target. Ther..

[180] Shi L., Yuan X., Yao W., Wang S., Zhang C., Zhang B., Song J., Huang L., Xu Z., Fu J.L. (2022). Human mesenchymal stem cells treatment for severe COVID-19: 1-year follow-up results of a randomized, double-blind, placebo-controlled trial. EBioMedicine.

[181] Grégoire C., Layios N., Lambermont B., Lechanteur C., Briquet A., Bettonville V., Baudoux E., Thys M., Dardenne N., Misset B. (2022). Bone Marrow-Derived Mesenchymal Stromal Cell Therapy in Severe COVID-19: Preliminary Results of a Phase I/II Clinical Trial. Front. Immunol..

[182] Xu X., Jiang W., Chen L., Xu Z., Zhang Q., Zhu M., Ye P., Li H., Yu L., Zhou X. (2021). Evaluation of the safety and efficacy of using human menstrual blood-derived mesenchymal stromal cells in treating severe and critically ill COVID-19 patients: An exploratory clinical trial. Clin. Transl. Med..

[183] Saleh M., Vaezi A.A., Aliannejad R., Sohrabpour A.A., Kiaei S.Z.F., Shadnoush M., Siavashi V., Aghaghazvini L., Khoundabi B., Abdoli S. (2021). Cell therapy in patients with COVID-19 using Wharton’s jelly mesenchymal stem cells: A phase 1 clinical trial. Stem Cell Res. Ther..

[184] Brizuela C., Meza G., Urrejola D., Quezada M.A., Concha G., Ramírez V., Angelopoulos I., Cadiz M.I., Tapia-Limonchi R., Khoury M. (2020). Cell-Based Regenerative Endodontics for Treatment of Periapical Lesions: A Randomized, Controlled Phase I/II Clinical Trial. J. Dent. Res..

[185] Gratwohl A., Passweg J., Gerber I., Tyndall A. (2001). Stem cell transplantation for autoimmune diseases. Best. Pract. Res. Clin. Haematol..

[186] Sykes M., Nikolic B. (2005). Treatment of severe autoimmune disease by stem-cell transplantation. Nature.

[187] Yu L., Zeng L., Zhang Z., Zhu G., Xu Z., Xia J., Weng J., Li J., Pathak J.L. (2023). Cannabidiol Rescues TNF-α-Inhibited Proliferation, Migration, and Osteogenic/Odontogenic Differentiation of Dental Pulp Stem Cells. Biomolecules.

